# Early-stage cost-utility analysis of novel diagnostic tests for giant cell arteritis: a modelling study in UK secondary care

**DOI:** 10.1136/bmjopen-2025-102888

**Published:** 2025-11-13

**Authors:** Miaoqing Yang, Paola Cocco, Sarah L Mackie, Ann W Morgan

**Affiliations:** 1 School of Medicine, University of Leeds, Leeds, UK; 2 NIHR Leeds Biomedical Research Centre, Leeds Teaching Hospitals NHS Trust, Leeds, UK

**Keywords:** Rheumatology, HEALTH ECONOMICS, Rare Diseases

## Abstract

**Abstract:**

**Objective:**

To identify the key characteristics required for hypothetical diagnostic tests to be cost-effective for diagnosing giant cell arteritis (GCA).

**Design:**

Combined decision tree and Markov cohort state-transition models were used to evaluate the cost-utility of new diagnostic tests compared with the standard pathways of biopsy and clinical judgement, with and without ultrasound. Input parameters were derived from secondary data and expert opinions. The analysis adopted a lifetime horizon and the UK National Health Service (NHS) perspective, using a willingness-to-pay threshold of £20 000 per quality-adjusted life year (QALY). Bivariate deterministic sensitivity analyses identified the maximum test price at varying diagnostic performance levels, and probabilistic sensitivity analyses over 5000 simulations provided 95% CIs.

**Setting:**

UK.

**Participants:**

Patients with symptoms suggestive of GCA.

**Main outcome measure:**

Percentage of GCA-related and glucocorticoid-related complications avoided, maximum test price and incremental QALYs at each sensitivity and specificity combination.

**Results:**

A biomarker test incorporated into a hypothetical diagnostic pathway with perfect accuracy (100% sensitivity and specificity) can be priced up to £7245 (95% CI £5763 to £8727) and remain cost-effective compared with a standard pathway of temporal artery biopsy and clinical judgement. Against a standard pathway including ultrasound, the biomarker test can be priced up to £8606 (£6741 to £10 471). The test’s value was more strongly influenced by improvements in specificity than in sensitivity. The maximum prices decreased with earlier starting age, lower clinician adherence, shorter time horizons and shorter durations of glucocorticoid-related effects.

**Conclusions:**

The study highlights the potential for hypothetical tests to improve GCA diagnosis and reduce glucocorticoid toxicity, while demonstrating their market viability for use within the NHS. It also illustrates how early-stage economic models can provide valuable insights into potential cost-effectiveness to inform the test development process.

**STRENGTHS AND LIMITATIONS OF THIS STUDY**

- This is the first early economic evaluation to assess the potential cost-effectiveness of hypothetical biomarker tests for giant cell arteritis using decision-analytic modelling.
- Maximum cost-effective prices were estimated for each combination of sensitivity and specificity, providing actionable guidance for test developers on performance thresholds and pricing targets.
- The model assumed an average UK National Health Service (NHS) pathway, which may not capture variation across different NHS Trusts.
- Diagnostic tests were modelled as a bundled pathway rather than sequentially, simplifying interdependencies between test results.
- Gender-specific risks for adverse events and complications were not incorporated due to data limitations.

## Introduction

 Giant cell arteritis (GCA) is a critical ischaemic disease and the most prevalent form of systemic vasculitis. Its annual incidence is estimated to be approximately 2.2 per 10 000 person years in the UK.^1^ It is considered a medical emergency and characterised by inflammation of blood vessels, potentially leading to irreversible blindness if not promptly treated. Suspected cases typically arise in patients over 50 years old with new-onset symptoms such as headaches and temporal artery abnormalities, alongside fatigue, fever, weight loss and other varying symptoms dependent on the blood vessels involved.^2^ A patient with suspected GCA usually starts with high-dose glucocorticoid treatment (eg, prednisolone 40 mg daily, or 60 mg in the presence of ischaemic features), often before confirmation of diagnosis through further testing.^3^ While the treatment mitigates the risk of blindness, high-dose and long-term glucocorticoid use causes many side effects, including accelerated cardiovascular disease, diabetes, fractures and severe infections.^4^ Accurate and timely diagnosis therefore becomes crucial in determining the necessity of sustained high-dose glucocorticoid therapy to manage the condition effectively. Patients with suspected GCA should be referred to secondary care as quickly as possible for further investigations, which include (1) blood tests for the measurement of full blood count (platelets), erythrocyte sedimentation rate and C-reactive protein and (2) temporal artery biopsies (TAB) or ultrasound (US) as confirmatory tests.^2^ The gold standard diagnostic test in GCA is a TAB, a minor surgical procedure that involves obtaining a specimen from one of the arteries on the side of the head. While a positive TAB confirms GCA, negative results can occur in affected patients due to delays in performing the biopsy after initiation of treatment, patchy arterial involvement (skip lesions) or suboptimal biopsy techniques.^5 6^ In recent years, US has emerged as a non-invasive alternative diagnostic tool to TAB for GCA diagnosis. Evidence suggested that US is slightly more sensitive but less specific than TAB.^3^ By providing quicker access to confirmatory testing, US enables more rapid diagnosis and can also be used sequentially with TAB to enhance overall diagnostic accuracy. US is now available in most UK hospitals, although its accuracy depends on both the experience of the individuals who conduct the scans and the timing of the diagnosis. Since the characteristic halo sign on US diminishes after glucocorticoid initiation, imaging is recommended within 72 hours of starting glucocorticoid therapy.^6 7^ In contrast, TAB remains positive for up to 4 weeks but is often delayed, reducing its diagnostic yield.

There is a need to develop new tests for GCA that can accelerate diagnosis, improve accuracy and minimise glucocorticoid toxicity in individuals without GCA. New tests, however, are often expensive and time consuming to develop; therefore, it is important for developers to prioritise the ones that are likely to be cost-effective at an early stage. The goal of health economic modelling is to evaluate the costs and clinical benefits of a new intervention compared with standard care. Increasingly, these methods are being applied early in test development to help select promising candidates for further research and development (R&D).^8^ Economic modelling could provide an initial assessment of whether a new test could be cost-effective, and under which assumptions, before significant resources are invested.^9^ Even without diagnostic performance data, it can estimate the improvement in diagnostic accuracy from the current practices and incorporate potential downstream impact on both clinical outcomes and healthcare costs.

We performed an early-stage economic evaluation to assess the potential cost-effectiveness of hypothetical biomarker tests for diagnosing GCA within the National Health Service (NHS) in England. To inform the structure and inputs for our model, we drew on the Temporal Artery Biopsy and Ultrasound in Diagnosis of Giant Cell Arteritis (TABUL) study, the only UK-based economic evaluation to date that assessed the cost-effectiveness of diagnostic strategies for suspected GCA within the NHS, comparing US with TAB.^10^ Our study adapted the TABUL model to an early economic evaluation framework, estimating the maximum price at which biomarker tests could remain cost-effective compared with current practice, across a range of diagnostic sensitivity and specificity values (the ability of a test to correctly identify those with and without the disease). While molecular biomarker tests are one example of innovations that could reduce diagnostic delays and enhance GCA management, other test candidates, such as MRI, are also under consideration. Our analysis provides a framework that could be applied to evaluate a range of new diagnostic tests, offering valuable insights for developers when deciding whether to pursue new tests for GCA diagnosis.

## Methods

### Model overview

We conducted an early-stage cost-utility analysis evaluating the potential economic value of a series of biomarker tests and assessing their performance against the standard diagnostic pathway currently used in clinics. To inform the development of our model, we reviewed existing economic literature and found no prior economic evaluations of biomarker tests for diagnosing GCA. The only published economic evaluation of diagnostic strategies for suspected GCA is the TABUL study, which compared US with TAB in a UK NHS setting.^10^ We adapted some elements of the TABUL model, specifically the structure for long-term extrapolation for GCA-related complications and glucocorticoid-related adverse events and diagnostic performance data for the standard test pathway. Other input parameters were sourced from published literature and expert opinion, with priority given to studies providing distributions for probabilistic sensitivity analysis (PSA). Where multiple sources were available, selection was made through consensus among clinical experts (AWM and SLM). Reasonable assumptions were applied where data were unavailable.

The starting population comprised patients aged 71 years (the median age of the UK GCA population) with suspected GCA in secondary care. We employed a lifetime horizon of 30 years, assuming no individuals live beyond age 100. The model was developed and analysed in accordance with the guidelines outlined in the National Institute of Health and Care Excellence reference case.^11^ Our analysis adopted the NHS and Personal Social Services (PSS) perspective, which includes costs borne by the NHS and local authorities for delivering health and social care services.^11^ Both costs and benefits are discounted at a rate of 3.5% per annum. All costs are denominated in UK pounds sterling (£) at 2022/2023 values, based on the pay and prices index from the PSS Research Unit, which publishes annually updated unit costs estimates widely used in UK health economic evaluations.^12^ Our findings were presented as the incremental quality-adjusted life years (QALYs) and maximum price of hypothetical biomarker tests at each combination of sensitivity and specificity improvements compared with the standard test pathway. We employed a willingness-to-pay threshold of £20 000 per QALY. To ensure the model’s accuracy and relevance, our model was also reviewed and validated by clinical experts (AWM and SLM), with modifications made as necessary. The model was constructed and analysed using the R Studio V.2024.12.1 and following the Decision Analysis in R for Technologies in Health (DARTH) framework.^13 14^ A health economic analysis plan was developed and is available upon request.

### Model structure

**Figure 1:**
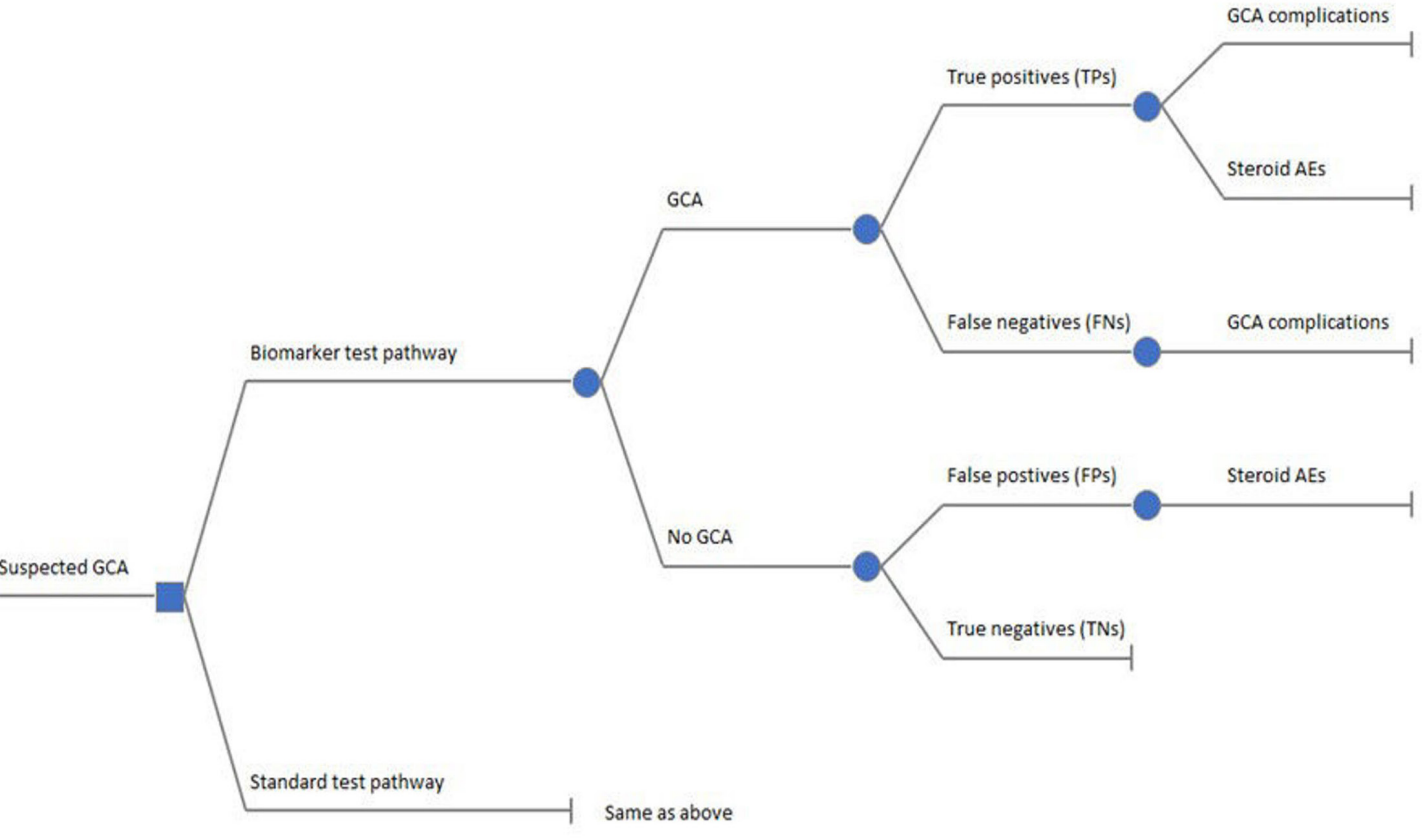
Decision tree model for diagnostic tests. AE, adverse events; GCA, giant cell arteritis.

**Figure 2:**
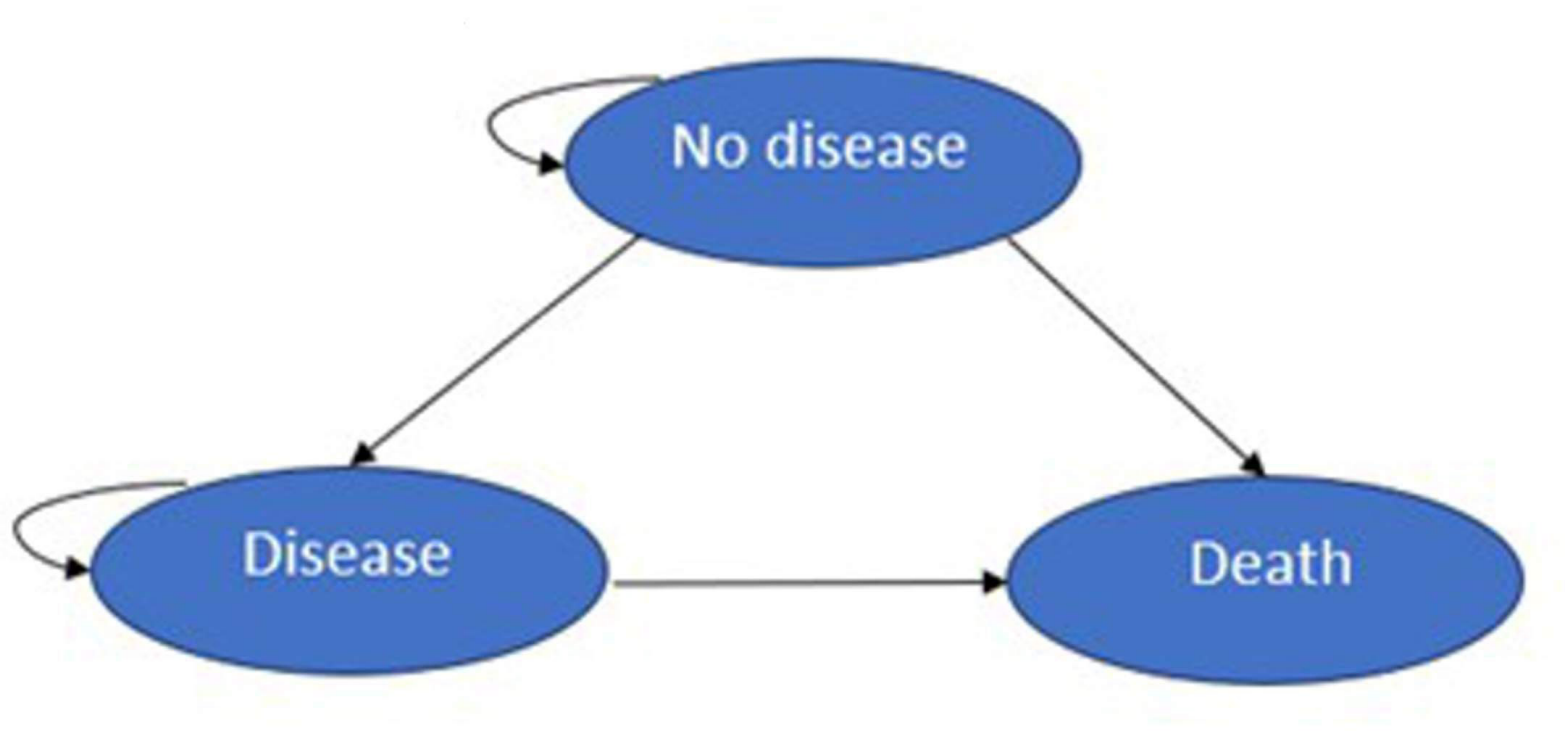
State-transition models for GCA complications and glucocorticoid-related adverse events. GCA, giant cell arteritis.

The model structure consisted of a decision tree for the diagnostic part (figure 1) followed by seven state-transition models (figure 2) to simulate the long-term health and economic consequence. The following sections describe the main components of the model structure.

#### Decision tree

The decision tree model was used to evaluate the cost-effectiveness of the new diagnostic pathway incorporating biomarker tests compared with standard care. The new biomarker tests were assessed as supplementary add-on tests incorporated into the standard blood test protocol, preceding the utilisation of TAB (and US) as confirmatory tests. We combined all tests into a unified assessment of overall diagnostic performance, as clinicians make decisions based on all the information available to them at the point in time, including symptoms, physical signs, results from laboratory tests and further diagnostic procedures such as TAB and US.^15^ Hence, our analysis focused on the overall test accuracy in terms of sensitivity and specificity values.

Each patient is assigned a test result depending on the diagnostic option being evaluated, including:

- True positive (TP)—indicating GCA presence and positive test results.
- True negative (TN)—signifying the absence of GCA and negative test results.
- False positive (FP)—signifying the absence of GCA yet positive test outcomes.
- False negative (FN) —indicating GCA presence despite negative test results.

Individuals presenting with symptoms suggestive of GCA and testing positive (ie, TPs and FPs) are given glucocorticoid treatment, exposing them to the risk of developing glucocorticoid-related adverse events. Individuals who have GCA (ie, TPs and FNs) are faced with the risk of developing GCA-related complications (eg, vision loss and stroke). The long-term impact of diagnostic strategies on costs and QALYs related to glucocorticoid-induced adverse events and GCA-related complications was derived using a series of state-transition models described below.

#### Markov cohort state-transition models

We employed a series of time-dependent state-transition models to extrapolate outcomes beyond the diagnostic endpoints of the decision tree. Each model for a specific condition comprised three states: no disease, disease and death. Transition probabilities for glucocorticoid-related adverse events were derived from risk data obtained from studies based on individual patient-level data from the Clinical Practice Research Datalink (CPRD).^16^,^19^ The risk of having adverse events varied based on both current and cumulative prednisolone equivalent dosages; for instance, some conditions (like fractures) are mainly influenced by cumulative glucocorticoid dosages in the long term, while others (such as myocardial infarction (MI) and stroke) are affected by current high glucocorticoid dosages. We therefore employed monthly cycles for the first 2 years to capture the effects of both current and cumulative prednisolone equivalent dosages on costs and QALYs, reflecting the typical tapering period of approximately 19 months recommended in the 2020 British Society for Rheumatology (BSR) guideline for GCA management.^20^ This involved calculating the cohort’s costs and QALYs proportionate to either remaining in the no disease state or transitioning to a disease state or death. We then switched to yearly cycles to estimate long-term costs and QALYs over the model time horizon. The per-patient total costs and QALYs for each GCA-related complication and glucocorticoid-induced adverse event were aggregated for TPs, FNs, FPs and TNs, respectively. These values were then used as endpoints in the decision tree analysis, enabling a comparison of the costs and QALYs of hypothetical biomarker tests with those of the current diagnostic pathway.

### Model assumptions

The following assumptions were made to enable the analysis to be carried out:

1. All FNs were assumed to have symptom recurrence within a short timeframe and re-enter the healthcare system for further consultation and retesting. Following the assumptions made in the TABUL analysis, we assumed that 25% of them were detected in month 2, 50% in month 3 and the remaining cases by month 4. The results from retesting were considered independent of the initial test results.
2. We assumed that clinicians adhere strictly to diagnostic results and glucocorticoid use guidelines, and all patients accept the tests and treatments offered. This assumption was relaxed in scenario analyses by allowing a proportion of TNs to be treated as TPs, reflecting scenarios where clinicians may override test results based on clinical judgement.
3. Due to scarce data on risk estimates for developing multiple long-term conditions, we treated GCA-related and glucocorticoid-related complications as independent from each other.
4. We assumed that new cases of vision loss were limited to the first year, given that the risk declines significantly after the initiation of glucocorticoid treatment. For other glucocorticoid-related conditions, new incidences were assumed to occur within the first 5 years. As we did not have data to account for how long adverse event risks persist after treatment cessation or whether they decline over time, we varied the duration of steroid-related impacts on costs and QALYs in scenario analyses to explore the influence of this uncertainty.
5. Despite the higher prevalence of GCA in women, we assumed uniform risk estimates for glucocorticoid-related adverse events and GCA-related complications across genders due to the lack of gender-specific data.

### Input parameters

A variety of secondary sources were used to parameterise the model, identified through previous literature and consultations with clinical experts (AWM and SLM). Table 1 presents the input parameter values and their sources, with further details provided in the sections below.

**Table 1:** Input parameters

Description	Mean (SE)	Source
Proportion of the true GCA among people with suspected GCA in secondary care	0.29*	Rajeswaran *et al*^21^
Sensitivity of TAB and clinical judgement	0.91 (0.018)	Luqmani *et al*^10^
Specificity of TAB and clinical judgement	0.81 (0.038)	Luqmani *et al*^10^
Sensitivity of TAB plus US and clinical judgement	0.96 (0.013)	Luqmani *et al*^10^
Specificity of TAB plus US and clinical judgement	0.77 (0.041)	Luqmani *et al*^10^
Cost of TAB	1872 (187.2)	NHS reference cost 2022/23^31^: YQ43Z day case
Cost of US	125 (12.5)	NHS reference cost 2022/23^31^ RD42Z
Total cost of glucocorticoid treatments for TPs and FPs	51.51 (5.2)	BNF^26^ accessed 18/06/2024
Total cost of glucocorticoid treatments for TNs and FNs	10.92 (1.09)	BNF^26^ accessed 18/06/2024
Baseline utility	0.799 (-)†	Alava *et al*^32^
Cost of symptom reappearance for FNs	205.5 (20.55)	Jones *et al* (PSSRU unit costs)^12^ and NHS reference cost 2022/23^31^
Utility of symptom reappearance for FNs	0.53 (0.025)	Luqmani *et al*^10^
Risk of vision loss among FNs	0.132 (0.338)	Chaddock *et al*^24^
Risk of vision loss among TPs	0.0099 (0.0003)	Luqmani *et al*^10^ and Niederkohr *et al*^23^
Annual costs of vision loss in the first year	5589 (558.9)	Colquitt *et al*^27^ and Hayreh *et al*^28^
Annual costs of vision loss from year 2 onwards	5384 (538.4)	Colquitt *et al*^27^ and Hayreh *et al*^28^
Overall utility value for vision loss	0.375 (0.039)	Brown *et al*^33^
Risk of cerebrovascular disease (stroke) among glucocorticoid non-users (for TPs, FPs and TNs)	0.007 (0.001)	Pujades-Rodrigue *et al*^17^
Risk of cerebrovascular disease (stroke) among glucocorticoid non-users (for FNs)	0.010 (0.101)	Chaddock *et al*^24^
Risk of MI among glucocorticoid non-users	0.006 (0.001)	Pujades-Rodrigue *et al*^17^
Risk of heart failure among glucocorticoid non-users	0.011 (0.002)	Pujades-Rodrigue *et al*^17^
Cost of cerebrovascular disease (stroke) in the first year	4962.42 (206.59)	Danese *et al*^37^
Cost of MI in the first year	5970.65 (178.76)	Danese *et al*^37^
Cost of heart failure in the first year	3498.25 (193.78)	Danese *et al*^37^
Cost of cerebrovascular disease (stroke) from year 2 onwards	520.835 (137.88)	Danese *et al*^37^
Cost of MI from year 2 onwards	493.958 (83.31)	Danese *et al*^37^
Cost of heart failure from year 2 onwards	453.860 (132.61)	Danese *et al*^37^
Utility of cerebrovascular disease (stroke) in the first year	0.646 (0.010)	Luengo-Fernandez *et al*^38^
Utility of MI in the first year	0.652 (0.007)	Pockett *et al*^39^
Utility of heart failure in the first year	0.57 (0.002)	Mejía *et al*^40^
Utility of cerebrovascular disease (stroke) from year 2 onwards	0.63 (0.012)	Luengo-Fernandez *et al*^38^
Utility of MI from year 2 onwards	0.654 (0.008)	Pockett *et al*^39^
Utility of heart failure from year 2 onwards	0.57 (0.017)	Mejía *et al*^40^
Risk of any fracture among glucocorticoid non-users	0.031 (0.005)	Wu *et al* (work in progress)^25^
Cost of any fracture in year 1	2357.60 (100.76)	Gutiérrez *et al*^30^ and Gutiérrez *et al*^29^
Cost of any fracture in year 2 and onwards	388.542 (98.176)	Gutiérrez *et al*^30^ and Gutiérrez *et al*^29^
Utility of any fracture in year 1	0.638 (0.033)	Ström *et al*^41^ and Zethraeus *et al*^42^
Utility of any fracture in year 2 and onwards	0.738 (0.014)	Ström *et al*^41^ and Zethraeus *et al*^42^
Cost of bone protection therapy: alendronate combined with calcium and vitamin D supplementation for primary prevention	63.96 (6.396)	BNF^26^ accessed on 06/08/2024
Risk of diabetes mellitus	0.009 (0.001)	Wu *et al*^19^
Cost of diabetes mellitus	558.48 (38.357)	Zhou *et al*^43^
Utility of diabetes mellitus	0.728 (0.048)	Sullivan *et al*^44^
Risk of hospitalised infection among glucocorticoid non-users	0.043 (0.0002)	Wu *et al*^18^
Cost of hospitalised infections	3074.22 (307.4)	NHS reference cost 2022/23^31^
Utility of hospitalised infection	0.609 (-)†	Niederkohr *et al*^23^

* As we did not have specific distribution parameters for GCA prevalence, we tested a lower proportion of 20% based on the discussions with the clinical experts in the sensitivity analyses. To derive the probabilistic distribution, we used the ‘*betaExpert*’ function from the R package *prevalence*.^45^ This function fits a Beta distribution using expert opinion, providing a best-guess estimate (mode or mean) and an uncertainty range from a specified lower bound.

† No SE value was found for the baseline utility; thus, use uniform distribution in the probabilistic sensitivity analysis.

BNF, British National Formulary; FNs, false negatives; FPs, false positives; GCA, giant cell arteritis; MI, myocardial infarction; NHS, National Health Service; PSSRU, Personal Social Services Research Unit; TAB, temporal artery biopsy; TNs, true negatives; TPs, true positives; US, ultrasound.

#### Prevalence of GCA

Based on local data from Leeds Teaching Hospitals NHS Trust,^21^ where TAB was used as the reference standard, the prevalence of GCA in the base case was assumed to be 29%. This value is lower than the prevalence reported in the TABUL study (262 out of 381), likely because the TABUL trial targeted patients whom clinicians identified as needing urgent TAB, leading to the exclusion of lower-risk cases. In contrast, our model aimed to cover a broader population, including lower-risk patients, to better represent the real-world scenarios of individuals with suspected GCA in secondary care.

#### Accuracy of standard test pathway

The diagnostic sensitivity and specificity of the standard test pathway (‘Two-week decision: TAB and clinical judgment’ and ‘Two-week decision: TAB and US and clinical judgement’) were obtained from the TABUL study, which were based on the rheumatologist’s interpretation of TAB (and US) findings at 2 weeks.^10^ We chose strategies involving clinical judgement from rheumatologists, aligning with current practice where decisions are informed by disease history, blood test results and response to high-dose glucocorticoid therapy, in conjunction with TAB or US test outcomes. Strategies incorporating risk stratification were not chosen due to the lack of specific guidelines in the current care pathway. Additionally, ‘Two-week decision: US and clinical judgment’ was not selected as the standard care since its performance data were derived from a vignette exercise rather than actual trial data. This limitation arose because US results were only provided in the TABUL trial if clinicians intended to rapidly reduce glucocorticoid doses at 2 weeks following a negative TAB and clinical evaluation.

#### Transition Probabilities

The risk of vision loss was estimated at 13% for FNs, where 195 out of 1478 individuals experienced permanent vision loss.^22^ For TPs, we followed the TABUL study, applying a 1.3% risk of occurrence of initial vision and excluding 24% of patients who avoided blindness after timely glucocorticoid treatment.^23^ The risk of stroke among FNs was informed by analysis using individual-level data from the UKGCA Consortium, which identified 20 cases with a cerebrovascular accident at diagnosis considered to be secondary to GCA out of 1946 individuals.^24^

The baseline risk of glucocorticoid-related adverse events among GCA patients not using glucocorticoids was based on the CPRD analyses mentioned above.^17^,^19^ Note that for infections, we only accounted for those leading to hospital admissions. Among the 22 234 patients who had an infection, 5937 (26.7%) were admitted to the hospital on the date of or within 7 days after the infection diagnosis.^18^ The baseline risk of infection was calculated using the incidence of all-cause infections multiplied by the proportion of cases resulting in hospital admissions.

We then applied uplift HRs to the incidence of adverse outcomes among glucocorticoid users, accounting for current and cumulative prednisolone equivalent dosages. These HRs were also sourced from the CPRD analyses using estimates specific to the GCA population (online supplemental Table A1).^17^,^19^ Cardiovascular events were limited to stroke, heart failure and MI, as these conditions demonstrate a strong glucocorticoid dose-response relationship and have a significant impact on costs and QALYs. While the TABUL study considered stroke only as GCA complication, we believe its risk is also influenced by glucocorticoid use. Instead of categorising fractures into different types, we used the overall risk of any fracture, as HRs were only available for the occurrence of any fracture among individuals with GCA and/or polymyalgia rheumatica (PMR) in the publicly accessible data from the referenced publication.^25^

Baseline mortality in the general population was obtained from National Life Tables provided by the Office for National Statistics,^22^ using mortality data from 2018 to 2020, prior to the COVID-19 pandemic to avoid inflated mortality rates. To better reflect the gender distribution of the GCA population, we calculated a weighted average mortality rate using the proportion of females (72%) and males (28%) observed in the TABUL cohort.^10^ Excess mortality associated with GCA was derived from another CPRD analysis examining mortality in individuals with chronic inflammatory diseases who had long-term use of glucocorticoids, varying by both current and cumulative prednisolone dosages.^16^ Due to a lack of robust data on event-specific case fatality, we did not explicitly model disease-specific mortality risks and instead applied all-cause mortality rates throughout the Markov models.

#### Costs

##### Glucocorticoid costs

We used the drug tariff price for 5 mg, 2.5 mg and 1 mg prednisolone from British National Formulary.^26^ Unlike the TABUL study, we did not use cost for 25 mg tablets as they are rarely used in clinical practice and are much more expensive than 5 mg tablets. The total glucocorticoid cost was calculated based on the glucocorticoid dosing schedule for GCA from the 2020 BSR guideline, which recommends tapering and discontinuation within 19 months with sustained disease control and no relapse.^20^ The initial prednisolone dose is typically 60 mg daily for patients with ischaemic complications and 40 mg for others. For simplicity, we assumed a starting dose of 50 mg daily for the first 4 weeks, aligning with the average waiting time for a TAB examination. Unlike the TABUL study, which reduced prednisolone dosage for FPs after week 6, our model assumed that FPs follow the same tapering schedule as TPs, as FPs are rarely identified in real-world clinical practice. If a clinician believes a patient does not have GCA, glucocorticoids are tapered more quickly, based on the duration of initial glucocorticoid treatment. If used for less than 4 weeks, they can be stopped immediately, whereas treatment lasting over 4 weeks requires gradual tapering over 6–10 weeks. Since most patients receive glucocorticoids for more than 4 weeks before getting confirmatory test results and a clinic review, our model assumed a tapering period of about 10 weeks.

##### Costs of GCA-related and glucocorticoid-related complications

The cost of vision loss included one-off expenses (ie, blind registration, low-vision aids, low-vision rehabilitation) and annual costs (ie, community care, residential care, depression, and fall-related hip replacement due to visual impairment).^27^ Following the TABUL analysis, we applied these costs only to visual acuity states worse than 6/60 m and used a weighted average of both costs based on the proportional occurrence of visual loss by severity taken from Hayreh *et al*,^28^ as shown in online supplemental Table A2. The cost of fractures was derived from two UK studies by Gutiérrez *et al*,^29 30^ which estimated the 1-year incremental costs for hip, vertebral and non-hip non-vertebral fractures, including costs associated with hospitalisations, general practice visits, accident and emergency (A&E) visits, referral visits and prescription medications. The year 1 costs used in our analysis match those from the TABUL study. To estimate the annual costs from year 2 onwards, we doubled the costs reported for days 181–365 in these studies. Since we only had risk data for any type of fracture, the costs and QALYs were weighted based on the proportions of people with each type of fracture.^25^ The cost of hospitalised infections was calculated as a weighted average of the total costs based on the proportions of different infection types, according to the NHS reference costs.^31^ These types included unspecified acute lower respiratory infections with interventions, gastrointestinal infections with multiple interventions and kidney or urinary tract infections, with or without interventions.

##### Costs of symptom recurrence

The TABUL analysis addressed symptom recurrence among FNs at a rate of 50 out of 57, assuming no impact on cost. In contrast, we assumed that all FNs would experience symptom recurrence and be detected by month 4 (see model assumption 1), receiving additional consultations and tests. For consultation costs, we included an extra general practitioner (GP) appointment^12^ and a consultant-led ophthalmology or rheumatology visit. The latter was calculated as a weighted average of consultant-led face-to-face visits (first or follow-up) for ophthalmology or rheumatology services, based on NHS reference cost activity data.^31^ While some FNs might seek emergency care, we did not include these costs due to their high variability and the lack of NHS reference costs for specific type of emergency services. Therefore, our model accounted only for GP and consultant-led care. FNs were also assumed to undergo another round of TAB or US testing.

### Utilities

#### Baseline utility

Baseline utility values for the general population were derived from a three-component adjusted limited dependent variable mixture model that analysed the EuroQol five-dimension three-level questionnaire (EQ-5D-3L) data from the 2014 wave of Health Survey for England dataset.^32^ We took the average utility for males and females aged above 70 and used 0.799 as the baseline utility for the general population in our model.

#### Utility of vision loss

Utility values for vision loss were derived from a cross-sectional study^33^ using time trade-off methods and followed the same weighted approach as the cost calculations.

#### Utility of symptom recurrence

Unlike the TABUL study, which applied a utility decrement for FNs experiencing symptom recurrence, we did not use this value due to its unclear source. Instead, QALY loss for FNs in our model was attributed solely to their increased risk of GCA-related complications (ie, vision loss, stroke).

### Analysis and dealing with uncertainty

We estimated the maximum cost-effective price (headroom) by comparing the standard test pathways with hypothetical, perfectly accurate (100% sensitivity and specificity) test pathways incorporating biomarker tests. The potential health gains were monetised using a willingness-to-pay threshold of £20 000 per QALY, estimating the headroom for biomarker tests at which a perfect test pathway could remain cost effective. Recognising that the new test pathways are unlikely to achieve perfect accuracy, we conducted bivariate deterministic sensitivity analysis (DSA), simultaneously varying sensitivity and specificity. Diagnostic performance improvements were divided into 10 equal increments, with starting points set either at the performance of the standard test pathways or at 0%. This approach allowed us to explore trade-offs between sensitivity and specificity, such as scenarios where new tests might achieve higher sensitivity but lower specificity, or vice versa. The diagnostic performance of the standard pathway was also included among the increment points to serve as a reference for current practice. For each pair of diagnostic performance levels, we calculated the percentage of GCA-related and glucocorticoid-related complications avoided, incremental QALYs gained and the maximum price at which the test would remain cost-effective.

For each uncertain parameter, a probability distribution was assigned: beta distributions for probability values and utilities and gamma distributions for costs. For cost values sourced from NHS reference costs, we assumed a SD equal to 10% of the mean value. The PSA involved sampling from the assigned distributions for each parameter. The model was run 5000 times, with each iteration randomly selecting parameter values from their respective distributions. The mean and 95% CI of the maximum costs and incremental QALYs were calculated by summarising the results across all the simulations.

In the base case analysis, we adopted a 30-year lifetime horizon and assumed that new glucocorticoid-related complications would develop only within the first 5 years (see model assumption 4). We conducted scenario analyses to explore parameter uncertainty, including varying the time horizon (5, 10 and 20 years), adjusting the duration of glucocorticoid-related condition impacts on costs and QALYs (2 and 10 years) and testing an alternative starting age of 51 years to reflect the lower bound of GCA onset. We also examined reduced clinician adherence to test results by assuming that 50% and 75% of TNs were treated as TPs (see model assumption 2). This reflects the most policy-relevant scenario, as TNs may be unnecessarily treated in the real-world clinical practice. Non-adherence in TPs and FNs was not modelled, as TPs are typically treated following a positive biopsy result, and FNs are often identified within a short timeframe, limiting their impact on long-term outcomes.

### Patient and public involvement and engagement

We engaged a diverse group of stakeholders throughout the development of this project, including clinical experts in rheumatology and histopathology, laboratory scientists, health economists, industry partners, and people with lived experience of GCA and policy makers from around the UK. A virtual workshop hosted by the NIHR Leeds In Vitro Diagnostic Co-Operative on 2 July 2021 was used to systematically explore unmet clinical needs in the diagnosis of GCA, guided by the European Federation of Clinical Chemistry and Laboratory Medicine Test Evaluation Working Group checklist.^34^ Two rheumatologists (AWM and SLM), who are also members of the research team and coauthors, contributed extensively across all stages of the project, including pathway mapping and interpretation of findings. Additionally, consultations with other clinicians were conducted during the development of the economic model. Their insights shaped key aspects of the model, including its structure, treatment pathways and the interpretation and presentation of findings.

## Results

**Figure 3:**
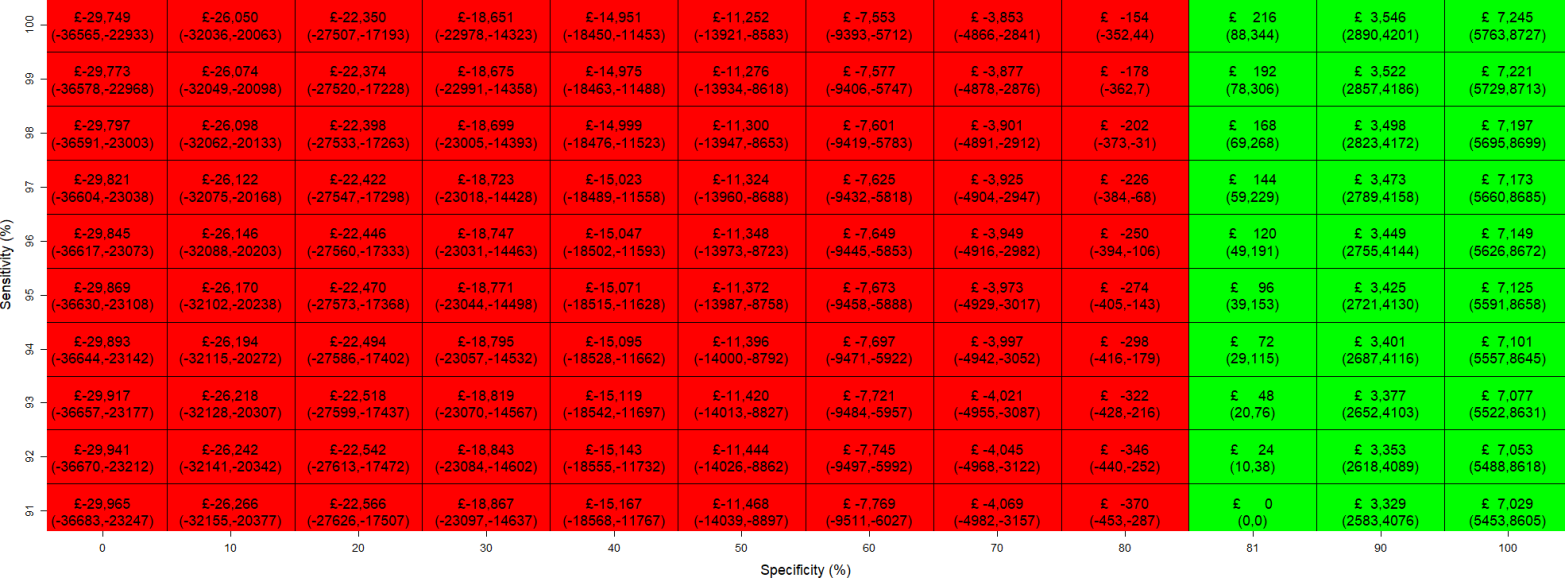
Maximum price at which the biomarker test is cost-effective at each diagnostic sensitivity (91–100%) and specificity (0–100%) pair for the biomarker test versus standard test pathway of TAB and clinical judgement. TAB, temporal artery biopsies.

**Figure 4:**
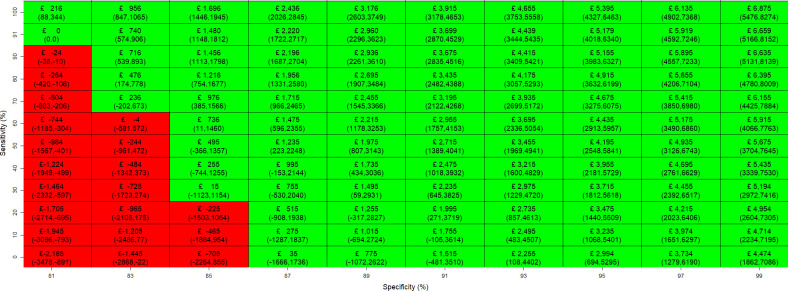
Maximum price at which the biomarker test is cost-effective at each diagnostic sensitivity (0–100%) and specificity (81–99%) pair for the biomarker test versus standard test pathway of TAB and clinical judgement. TAB, temporal artery biopsies.

Figures3  4 illustrate the maximum price of a biomarker test across combinations of sensitivity and specificity, compared with the standard test pathway of TAB and clinical judgement. The 95% CIs, shown in parentheses, were derived from PSA. In figure 3, sensitivity was varied from the performance level of the standard test pathway (91%) to 100%, while specificity ranged from 0% to 100%, with 81% included as the reference point for the specificity of the standard pathway. Figure 4 varied specificity from the standard test pathway level (81%) to 99%, with sensitivity ranging from 0% to 100%, and 91% included as the reference point for the sensitivity of the standard pathway.

A biomarker test incorporated into a perfect diagnostic pathway with overall sensitivity and specificity of 100% can be priced up to £7245 (95% CI £5763 to £8727) and remain cost-effective. When the diagnostic performance of the biomarker test pathway matches that of the standard test pathway (sensitivity 91% and specificity 81%), the maximum cost-effective price is zero. From this baseline, each 1% increase in specificity raises the maximum price by £370, whereas each 1% increase in sensitivity increases it by only £24. Therefore, improvements in test specificity have a larger impact on the maximum price. Notably, the biomarker test can still yield a positive cost-effective price even when sensitivity falls below 91%, provided that specificity exceeds the standard pathway level of 81%. However, if specificity falls below 81%, improvements in sensitivity alone are insufficient to generate a positive price, indicating that the test would not be cost-effective under those conditions. As shown in figure 4, when specificity reaches 87%, the biomarker test becomes cost-effective at any level of sensitivity. This pattern is also reflected in the incremental QALY gains shown in online supplemental Figures B1a and B1b, which illustrate the potential health benefits of introducing a biomarker test across varying levels of diagnostic accuracy. Incremental QALYs are mostly positive when specificity exceeds 81%, except when sensitivity is very low. Conversely, even at higher sensitivity levels, incremental QALYs turn negative when specificity does not improve sufficiently. A similar observation applies to the comparison with the standard test pathway of TAB, US and clinical judgement, where the biomarker test can be priced up to £8606 (£6741 to £10 471). The figures for maximum price and incremental QALYs are shown in online supplemental Figures C1a, C1b, C2a and C2b.

Table 2 presents the percentage reductions in GCA-related and glucocorticoid-related complications across different combinations of sensitivity and specificity for the biomarker test pathway, compared with the standard pathway of TAB and clinical judgement. When sensitivity is fixed at the standard pathway level (91%), increasing specificity leads to a progressive reduction in glucocorticoid-related adverse events. At specificity levels below 81%, the biomarker test pathway results in more FPs, increasing the disease incidence (reflected by negative percentages). As specificity exceeds 81%, these percentages turn positive, indicating fewer unnecessary treatments and associated harms. In contrast, when specificity is fixed at 81%, improvements in sensitivity primarily reduce the risk of vision loss by lowering the number of FNs. A biomarker test with perfect accuracy (100% sensitivity and specificity) is projected to reduce vision loss by 0.063%, while also preventing up to 0.227% of diabetes, 0.101% of heart failure, 0.085% of MI, 0.141% of strokes, 0.067% of infection and 0.243% of fractures compared with the standard test pathway. Online supplemental Table C1 shows a similar pattern when compared with the standard test pathway of TAB, US and clinical judgement.

**Table 2:** Percentage of GCA-related and glucocorticoid-related complications being avoided at each sensitivity and specificity pair for the biomarker test versus standard test pathway of TAB and clinical judgement

Test sensitivity (%)	Test specificity (%)	Vision loss (%)	Diabetes (%)	Heart failure (%)	MI (%)	Stroke (%)	Infection (%)	Fractures (%)
91	0	0.000	−0.731	−0.321	−0.282	−0.468	−0.187	−0.828
91	10	0.000	−0.641	−0.281	−0.247	−0.410	−0.164	−0.726
91	20	0.000	−0.551	−0.241	−0.212	−0.352	−0.141	−0.623
91	30	0.000	−0.460	−0.202	−0.177	−0.295	−0.118	−0.521
91	40	0.000	−0.370	−0.162	−0.143	−0.237	−0.095	−0.419
91	50	0.000	−0.280	−0.123	−0.108	−0.179	−0.072	−0.317
91	60	0.000	−0.190	−0.083	−0.073	−0.121	−0.049	−0.215
91	70	0.000	−0.099	−0.044	−0.038	−0.064	−0.025	−0.112
91	80	0.000	−0.009	−0.004	−0.003	−0.006	−0.002	−0.010
91	90	0.000	0.081	0.036	0.031	0.052	0.021	0.092
91	100	0.000	0.172	0.075	0.066	0.110	0.044	0.194
0	81	−0.640	−0.561	−0.259	−0.188	−0.315	−0.230	−0.498
10	81	−0.570	−0.500	−0.230	−0.168	−0.280	−0.205	−0.443
20	81	−0.499	−0.438	−0.202	−0.147	−0.246	−0.180	−0.389
30	81	−0.429	−0.376	−0.173	−0.126	−0.211	−0.154	−0.334
40	81	−0.359	−0.315	−0.145	−0.105	−0.176	−0.129	−0.279
50	81	−0.288	−0.253	−0.117	−0.085	−0.142	−0.104	−0.224
60	81	−0.218	−0.191	−0.088	−0.064	−0.107	−0.078	−0.170
70	81	−0.148	−0.130	−0.060	−0.043	−0.073	−0.053	−0.115
80	81	−0.077	−0.068	−0.031	−0.023	−0.038	−0.028	−0.060
90	81	−0.007	−0.006	−0.003	−0.002	−0.003	−0.003	−0.005
100	81	0.063	0.056	0.026	0.019	0.031	0.023	0.049

GCA, giant cell arteritis; MI, myocardial infarction; TAB, temporal artery biopsy.

Scenario analyses have been conducted to explore the impact of key modelling assumptions on the maximum cost-effective price (headroom) of the biomarker test. In online supplemental Figures D1a and D1b, when the starting age was reduced to 51 years, reflecting the lower bound of typical GCA onset, the headroom compared with the standard pathway of TAB and clinical judgement decreased to £3150 (£2651 to £3650). Reducing clinician adherence to test results also lowered the headroom: to £3596 (£2911 to £4281) when 50% of TNs were treated as TPs in online supplemental Figures D2a and D2b and to £5421 (£4339 to £6502) under a 75% misclassification rate in online supplemental Figures D3a and D3b. Shorter time horizons reduced the headroom as well, with estimates of £6898 (£5490 to £8306) for a 20-year horizon in online supplemental Figures D4a and D4b, £4450 (£3559 to £5341) for a 10-year horizon in online supplemental Figures D5a and D5b and £1907 (£1548 to £2266) for a 5-year horizon in online supplemental Figures D6a and D6b. Varying the duration of glucocorticoid-related impacts had a substantial effect: shortening the duration to 2 years reduced the headroom to £3136 (£2508 to £3764) in online supplemental Figures D7a and D7b, while extending it to 10 years increased the headroom to £12 667 (£9815 to £15 519) in online supplemental Figures D8a and D8b.

## Discussion

### Principal findings

The estimated headroom price for a biomarker test is £7245 (£5763 to £8727) and £8606 (£6741 to £10 471), when compared with the standard pathways of TAB and clinical judgement, with and without US. The test’s value was more strongly driven by improvements in test specificity, as FPs would remain undetected, while FNs are usually identified within a month. The maximum price is higher when compared with TAB, US and clinical judgement pathway, as its lower specificity allows more room for improvement. Scenario analyses demonstrate that reduced clinician adherence to test results lowers the headroom, as fewer patients benefit from the biomarker test’s improved diagnostic accuracy when clinicians override test results with their own judgement. Shorter time horizons also reduce headroom, reflecting lower cumulative costs and QALY losses from glucocorticoid-related complications. In contrast, extending the duration of glucocorticoid impact increases headroom, highlighting the longer-term economic burden of these adverse events. Notably, lowering the starting age to 51 years reduced headroom, contrary to initial expectations that a longer model time horizon would improve the maximum price. This effect appears to be driven by lower baseline mortality at younger ages, which reduces the impact of excess mortality from steroid use and narrows QALY differences between FPs and TNs.

### Strengths and weaknesses

This is the first early economic evaluation study examining diagnostic strategies for GCA. We demonstrate how decision modelling can help test developers assess the potential value and commercial viability of diagnostic tests early in development. Given the high costs and risks associated with test development, especially for small biotech companies, early economic evaluations offer a valuable tool to assess cost-effectiveness before significant resources are invested. Developers can refine tests to better meet clinical needs or abandon those unlikely to be viable. Although early evaluations often rely on expert judgement due to data limitations, our findings suggest that multivariate sensitivity analyses can provide meaningful insights in the absence of specific test performance data. The model, automated in R, offers greater efficiency than previous Excel-based models. By combining bivariate DSA with PSA, we explore trade-offs between sensitivity, specificity and cost while accounting for uncertainty in model inputs, generating a realistic range of maximum test prices with 95% CIs. This makes the analysis more actionable for test developers.

Several key assumptions warrant further consideration. Due to the hypothetical nature of the test, we relied on expert opinions to define the clinical pathway, which may not fully capture variations in real-world practice across NHS Trusts but only represent an average scenario in the UK. Additionally, we modelled multiple diagnostic tests as a bundled pathway rather than sequentially, simplifying the interdependence of test results. Furthermore, we were unable to incorporate gender-specific risk estimates for glucocorticoid-related adverse events and GCA-related complications due to the lack of available data, which may affect the precision of our results given the higher prevalence of GCA in women.

### Implications and future research

Using a model-based economic evaluation, we demonstrate the potential value of incorporating a hypothetical biomarker test into the current diagnostic pathway for patients with suspected GCA. According to tariff data from the Schedule of Events Cost Attribution Tool,^35^ the estimated cost of each biomarker test is about £22, which is substantially lower than the headroom prices identified in our model. This reinforces the economic case for further development and investment. The introduction of such a test could enhance diagnostic accuracy and reduce the risk of glucocorticoid-related complications, including diabetes, infection and fractures.

Another important advantage of introducing a less invasive and faster biomarker test is its potential to improve access to confirmatory testing, particularly for patients who currently miss out on TAB due to limited availability or clinical uncertainty. Access to TAB and US varies widely across NHS Trusts, with delays of up to 77 days for TAB and 20 days for US in some regions.^36^ In addition, US findings can disappear within 48–72 hours after initiating glucocorticoid therapy, necessitating very rapid turnaround to be clinically useful. A rapid biomarker test could help overcome these barriers by enabling earlier diagnostic confirmation and allowing faster clinical decision making. Furthermore, the availability of a reliable rule-out biomarker test could reduce the number of TAB and US performed by helping to exclude GCA earlier in the diagnostic pathway. While our model did not explicitly incorporate time-to-test results, the potential for such a test to shorten unnecessary glucocorticoid exposure in patients later found not to have GCA further strengthens the clinical and economic rationale for investment in biomarker development.

Our analytical framework is also adaptable to other diagnostic tools, such as MRI, and can support test developers in assessing feasibility, refining test design and determining the broader clinical and economic impact of new technologies. As more performance data become available, future research could build on this framework to conduct more comprehensive analyses and provide robust evidence to decision makers on the economic value of these tests.

It is important to note that this study explored a single implementation scenario in which the biomarker test acts as an add-on to the standard blood test protocol for patients with suspected GCA. However, the clinical and economic impact of such a test may vary depending on how and where the test is used within the care pathway, whether as a replacement, triage tool or add-on, and the timing of its use (eg, in primary vs secondary care). Additionally, different target populations and inclusion criteria could influence both effectiveness and cost-effectiveness. For example, biomarker tests could serve diagnostic, prognostic, monitoring, stratification or toxicity prediction functions, each with distinct implications for clinical utility and value. Future modelling work could explore these alternative use cases to better capture the full range of potential benefits and implementation challenges.

Finally, healthcare organisations need to consider the broader resource implications of introducing new diagnostics, including investment in equipment and training. For example, improving and maintaining high diagnostic accuracy require ongoing training to ensure high levels of operator expertise, particularly for techniques such as ultrasonography. The modelling work presented here may assist decision makers in evaluating resource allocation in relation to GCA pathways.

## Conclusion

This study shows the potential of hypothetical biomarker tests to enhance GCA diagnosis while reducing the risks of glucocorticoid toxicity and highlighted their feasibility for clinical adoption within the NHS. The model can be applied not only to biomarker tests for GCA diagnosis currently in development but also to other types of diagnostic tests for GCA. The study highlights the importance of early economic evaluation in test development, providing information about the potential cost-effectiveness of new tests, even with limited available evidence.

## Supplementary material

10.1136/bmjopen-2025-102888 online supplemental file 1

## Data Availability

Data are available upon reasonable request.
