# Supplementary material for: Early-stage cost-utility analysis of novel diagnostic tests for giant cell arteritis: a modelling study in UK secondary care
Source: BMJ Open. 2025 Nov 13;15(11):e102888. doi: 10.1136/bmjopen-2025-102888 (PMC12625940; doi:10.1136/bmjopen-2025-102888)

## Definition of input parameters

**Table A1: Hazard ratios of the risk of glucocorticoid-related conditions associated with current and cumulative glucocorticoid dosages by month**

|  | **Hazard ratios with SE** | | | | | | | | | |
| --- | --- | --- | --- | --- | --- | --- | --- | --- | --- | --- |
|  | **TPs and FPs** | | | | | **TNs and FNs** | | | | |
| **Months** | **Mortality** | **Diabetes** | **CVD** | **Infection** | **Fractures** | **Mortality** | **Diabetes** | **CVD** | **Infection** | **Fractures** |
| 1 | 1.200(0.098) | 4.346(0.782) | 3.146(0.452) | 2.323(0.145) | 1.944(0.803) | 1.200(0.023) | 4.346(0.782) | 3.146(0.452) | 2.323(0.145) | 1.944(0.803) |
| 2 | 1.428(0.057) | 4.928(0.840) | 3.146(0.452) | 2.323(0.145) | 1.944(0.803) | 1.428(0.057) | 2.908(0.353) | 1.938(0.162) | 1.475(0.061) | 1.890(0.435) |
| 3 | 1.428(0.057) | 3.988(0.639) | 2.360(0.285) | 1.677(0.094) | 1.534(0.687) | 0.750(0.040) | 1.270(0.088) | 1.140(0.056) | 1.010(0.023) | 1.080(0.136) |
| 4 | 1.968(0.080) | 3.894(0.621) | 2.505(0.299) | 1.743(0.096) | 1.619(0.714) | 1.190(0.025) | 1.270(0.088) | 1.140(0.056) | 1.010(0.023) | 1.080(0.136) |
| 5 | 1.968(0.080) | 2.840(0.343) | 2.057(0.170) | 1.533(0.062) | 1.995(0.449) | 1.190(0.025) | 1.270(0.088) | 1.140(0.056) | 1.010(0.023) | 1.080(0.136) |
| 6 | 1.968(0.080) | 2.840(0.343) | 2.057(0.170) | 1.533(0.062) | 1.995(0.449) | 1.190(0.025) | 1.270(0.088) | 1.140(0.056) | 1.010(0.023) | 1.080(0.136) |
| 7 | 1.968(0.080) | 2.840(0.343) | 2.057(0.170) | 1.533(0.062) | 1.995(0.449) | 1.190(0.025) | 1.270(0.088) | 1.140(0.056) | 1.010(0.023) | 1.080(0.136) |
| 8 | 1.968(0.080) | 2.840(0.343) | 2.057(0.170) | 1.533(0.062) | 1.995(0.449) | 1.190(0.025) | 1.270(0.088) | 1.140(0.056) | 1.010(0.023) | 1.080(0.136) |
| 9 | 1.968(0.080) | 2.840(0.343) | 2.057(0.170) | 1.533(0.062) | 1.995(0.449) | 1.190(0.025) | 1.270(0.088) | 1.140(0.056) | 1.010(0.023) | 1.080(0.136) |
| 10 | 1.689(0.082) | 2.840(0.343) | 2.057(0.170) | 1.533(0.062) | 1.995(0.449) | 1.190(0.025) | 1.270(0.088) | 1.140(0.056) | 1.010(0.023) | 1.080(0.136) |
| 11 | 1.689(0.082) | 2.840(0.343) | 2.057(0.170) | 1.533(0.062) | 1.995(0.449) | 1.190(0.025) | 1.270(0.088) | 1.140(0.056) | 1.010(0.023) | 1.080(0.136) |
| 12 | 1.689(0.082) | 2.840(0.343) | 2.057(0.170) | 1.533(0.062) | 1.995(0.449) | 1.190(0.025) | 1.270(0.088) | 1.140(0.056) | 1.010(0.023) | 1.080(0.136) |
| 13 | 1.689(0.082) | 2.840(0.343) | 2.057(0.170) | 1.533(0.062) | 1.995(0.449) | 1.190(0.025) | 1.270(0.088) | 1.140(0.056) | 1.010(0.023) | 1.080(0.136) |
| 14 | 1.033(0.055) | 2.480(0.311) | 1.815(0.170) | 1.460(0.061) | 1.801(0.489) | 1.190(0.025) | 1.270(0.088) | 1.140(0.056) | 1.010(0.023) | 1.080(0.136) |
| 15 | 1.033(0.055) | 2.480(0.311) | 1.815(0.170) | 1.460(0.061) | 1.801(0.489) | 1.190(0.025) | 1.270(0.088) | 1.140(0.056) | 1.010(0.023) | 1.080(0.136) |
| 16 | 1.033(0.055) | 2.480(0.311) | 1.815(0.170) | 1.460(0.061) | 1.801(0.489) | 1.190(0.025) | 1.270(0.088) | 1.140(0.056) | 1.010(0.023) | 1.080(0.136) |
| 17 | 1.033(0.055) | 2.480(0.311) | 1.815(0.170) | 1.460(0.061) | 1.801(0.489) | 1.190(0.025) | 1.270(0.088) | 1.140(0.056) | 1.010(0.023) | 1.080(0.136) |
| 18 | 1.033(0.055) | 2.480(0.311) | 1.815(0.170) | 1.460(0.061) | 1.801(0.489) | 1.190(0.025) | 1.270(0.088) | 1.140(0.056) | 1.010(0.023) | 1.080(0.136) |
| 19 | 1.033(0.055) | 2.480(0.311) | 1.815(0.170) | 1.460(0.061) | 1.801(0.489) | 1.190(0.025) | 1.270(0.088) | 1.140(0.056) | 1.010(0.023) | 1.080(0.136) |
| 20 | 1.640(0.035) | 1.240(0.086) | 1.210(0.058) | 1.050(0.023) | 1.140(0.139) | 1.190(0.025) | 1.270(0.088) | 1.140(0.056) | 1.010(0.023) | 1.080(0.136) |
| 21 | 1.640(0.035) | 1.240(0.086) | 1.210(0.058) | 1.050(0.023) | 1.140(0.139) | 1.190(0.025) | 1.270(0.088) | 1.140(0.056) | 1.010(0.023) | 1.080(0.136) |
| 22 | 1.640(0.035) | 1.240(0.086) | 1.210(0.058) | 1.050(0.023) | 1.140(0.139) | 1.190(0.025) | 1.270(0.088) | 1.140(0.056) | 1.010(0.023) | 1.080(0.136) |
| 23 | 1.640(0.035) | 1.240(0.086) | 1.210(0.058) | 1.050(0.023) | 1.140(0.139) | 1.190(0.025) | 1.270(0.088) | 1.140(0.056) | 1.010(0.023) | 1.080(0.136) |
| 24 | 1.640(0.035) | 1.240(0.086) | 1.210(0.058) | 1.050(0.023) | 1.140(0.139) | 1.190(0.025) | 1.270(0.088) | 1.140(0.056) | 1.010(0.023) | 1.080(0.136) |
| 25 | 1.640(0.035) | 1.240(0.086) | 1.210(0.058) | 1.050(0.023) | 1.140(0.139) | 1.190(0.025) | 1.270(0.088) | 1.140(0.056) | 1.010(0.023) | 1.080(0.136) |

**Table A2: Costs and QALYs of vision loss by visual acuity in one eye**

| **Severity** | **Proportion** | **Utility** | **Cost in year 1** | **Cost in year 2 onwards** |
| --- | --- | --- | --- | --- |
| 6/15 – 6/21 | 13% | 0.88^1^ |  |  |
| 6/24 – 6/30 | 4% | 0.77^2^ |  |  |
| 6/60 – 6/120 | 6% | 0.65 | 6,814 | 6,564 |
| Counting Fingers (CF) | 21% | 0.47 | 6,814 | 6,564 |
| Hand Motion (HM) | 17% | 0.47^3^ | 6,814 | 6,564 |
| Light perception (LP) | 15% | 0.47 | 6,814 | 6,564 |
| No Light perception (NLP) in one eye | 24% | 0.37^4^ | 6,814 | 6,564 |
| ***Average weighted by incidence*** |  | 0.524 | 5,589 | 5,384 |

*Notes*: ^1^ Assumed it equals the top tertile of the utility values between perfect vision and 6/60 vision; ^2^ Assumed it equals the bottom tertile of the utility values between perfect vision and 6/60 vision; ^3^ Assumed it is the same as the utility value of LP; ^4^ Assumed it equals the midpoint of the utility values between LP and NLP.

## Incremental QALYs at varying sensitivity and specificity for the biomarker test vs. standard test pathway of TAB and clinical judgment

**Figure B1a: Incremental QALYs at each diagnostic sensitivity (91-100%) and specificity (0-100%) pair for the biomarker test vs. standard test pathway of TAB and clinical judgment**

**
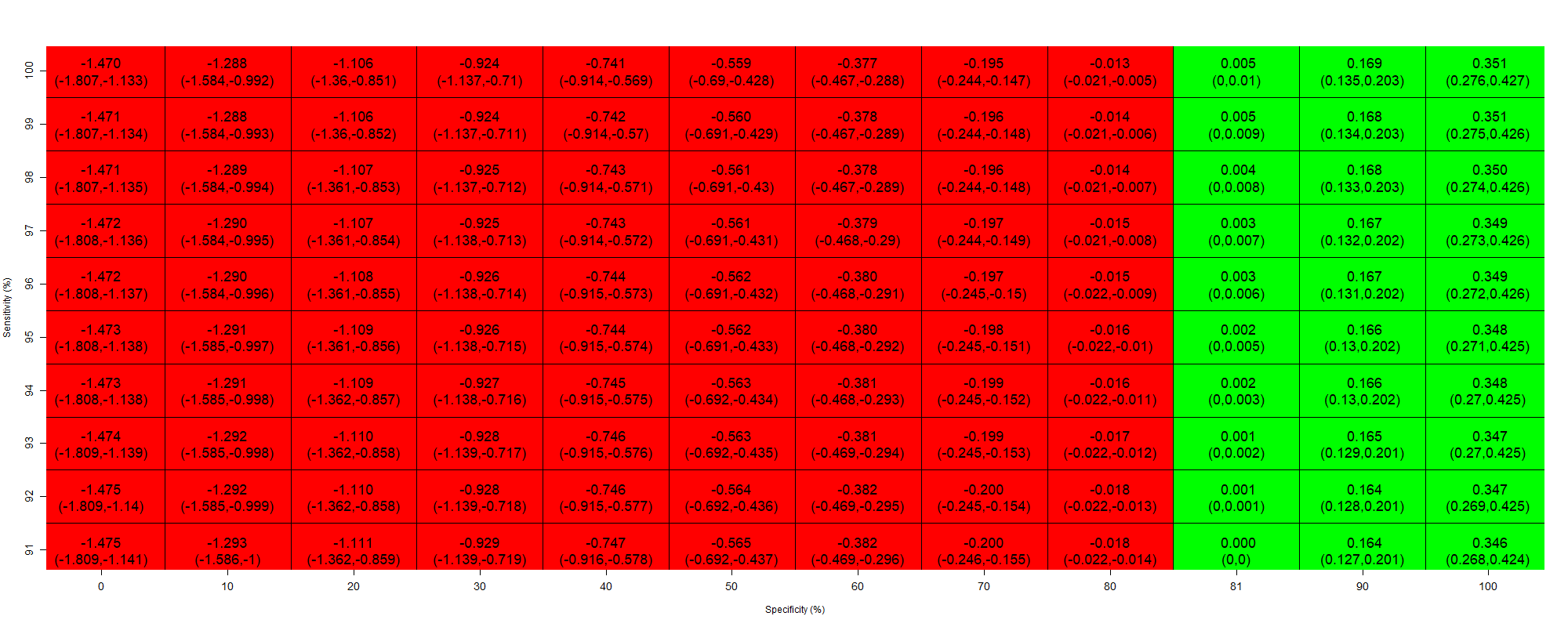
**

**Figure B1b: Incremental QALYs at each diagnostic sensitivity (0-100%) and specificity (81-99%) pair for the biomarker test vs. standard test pathway of TAB and clinical judgment**


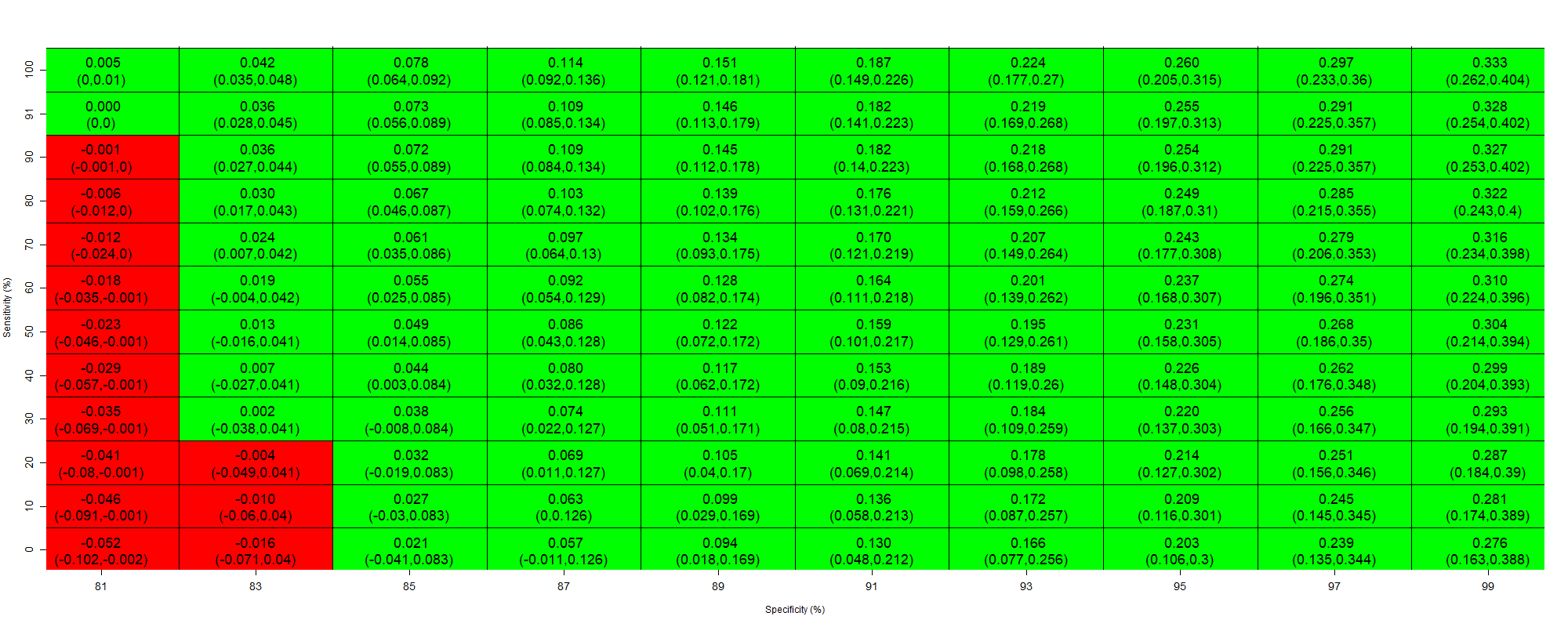


## Maximum prices and incremental QALYs at varying sensitivity and specificity for the biomarker test vs. standard test pathway of TAB and US and clinical judgment

**Figure C1a: Maximum price at which the biomarker test is cost-effective at each sensitivity (96-100%) and specificity (0-100%) pair for the biomarker test vs. standard test pathway** **of TAB and US and clinical judgment**


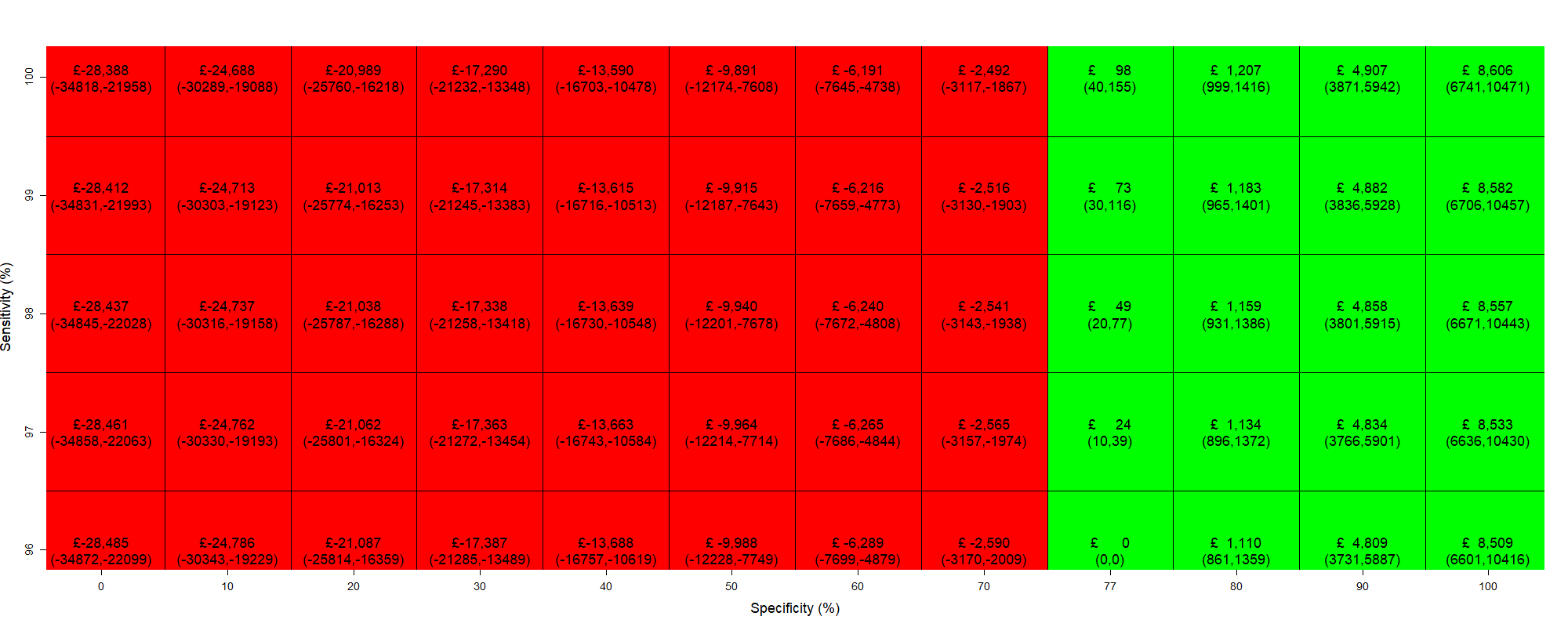


**Figure C1b: Maximum price at which the biomarker test is cost-effective at each sensitivity (0-100%) and specificity (77-99%) pair for the biomarker test vs. standard test pathway** **of TAB and US and clinical judgment**


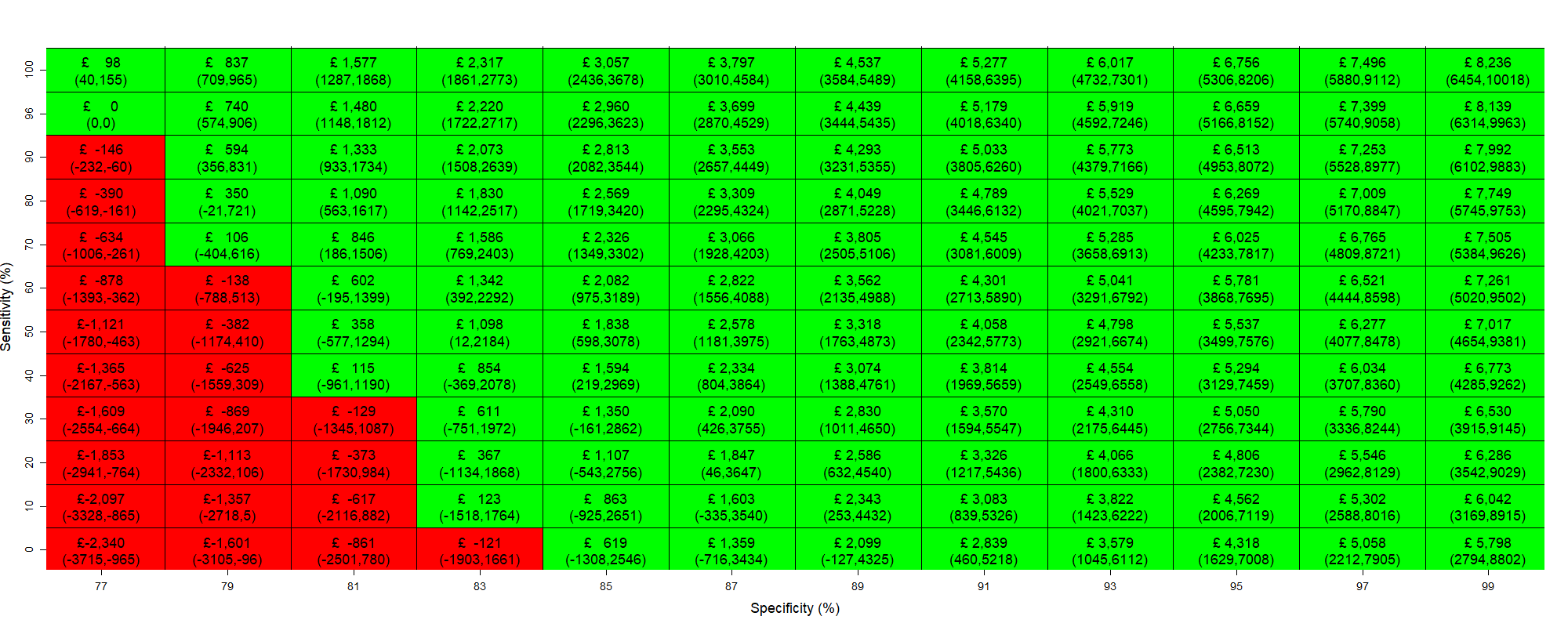


**Figure C2a: Incremental QALYs at each sensitivity (96-100%) and specificity (0-100%) pair for the biomarker test vs. standard test pathway** **of TAB and US and clinical judgment**


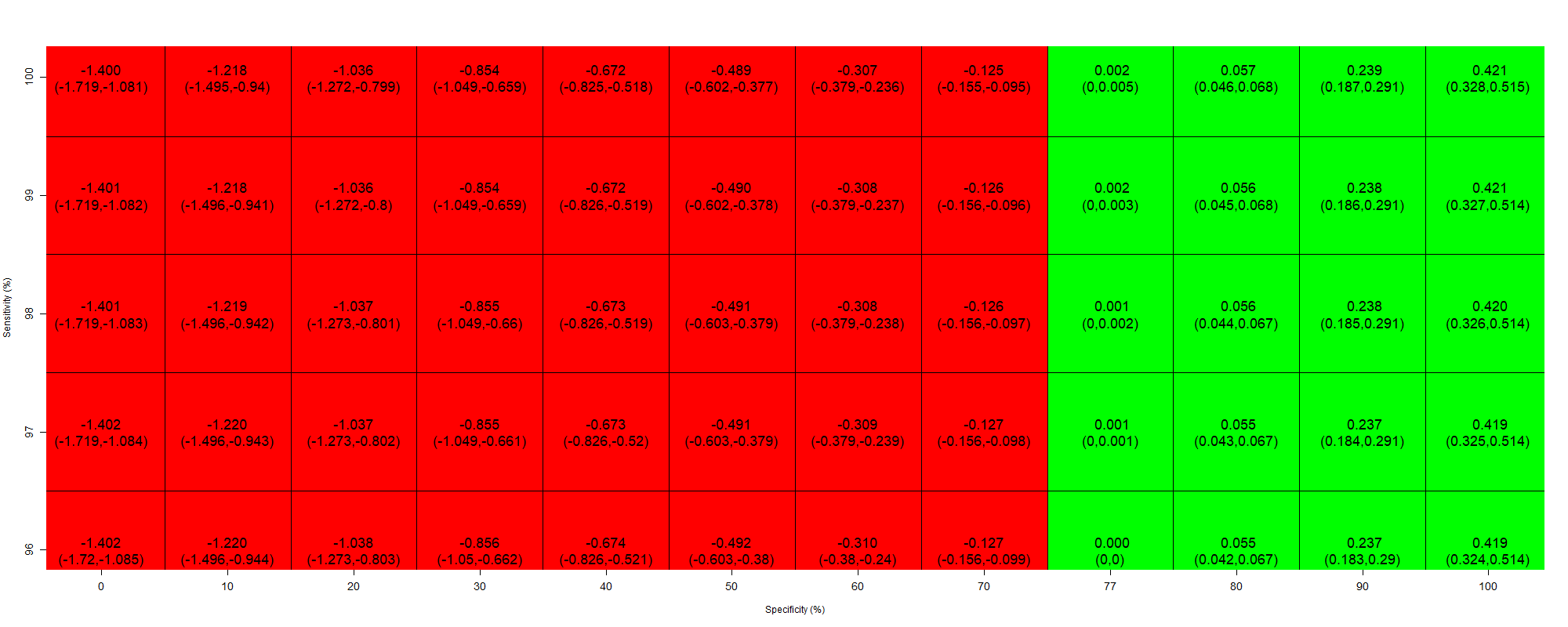


**Figure C2b: Incremental QALYs at each sensitivity (0-100%) and specificity (77-99%) pair for the biomarker test vs. standard test pathway** **of TAB and US and clinical judgment**


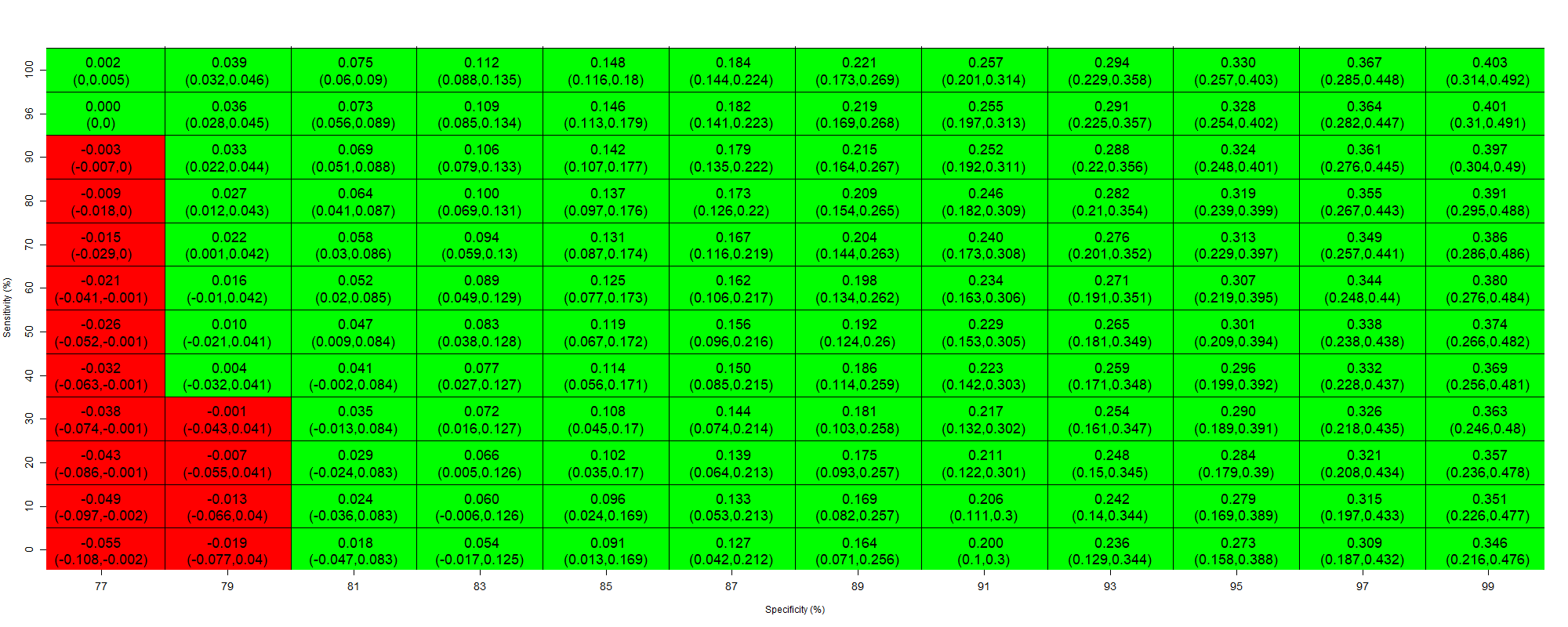


**Table C1: Percentage of GCA- and glucocorticoid-related complications being avoided at each sensitivity and specificity pair for the biomarker test vs. standard test pathway** **of TAB, US and clinical judgment**

| Test sensitivity (%) | Test specificity (%) | Vision loss (%) | Diabetes (%) | Heart failure (%) | MI (%) | Stroke (%) | Infection (%) | Fractures (%) |
| --- | --- | --- | --- | --- | --- | --- | --- | --- |
| 96 | 0 | 0.000 | -0.695 | -0.305 | -0.268 | -0.445 | -0.178 | -0.787 |
| 96 | 10 | 0.000 | -0.605 | -0.265 | -0.233 | -0.387 | -0.155 | -0.685 |
| 96 | 20 | 0.000 | -0.515 | -0.226 | -0.198 | -0.329 | -0.132 | -0.582 |
| 96 | 30 | 0.000 | -0.424 | -0.186 | -0.163 | -0.271 | -0.109 | -0.480 |
| 96 | 40 | 0.000 | -0.334 | -0.146 | -0.129 | -0.214 | -0.086 | -0.378 |
| 96 | 50 | 0.000 | -0.244 | -0.107 | -0.094 | -0.156 | -0.062 | -0.276 |
| 96 | 60 | 0.000 | -0.153 | -0.067 | -0.059 | -0.098 | -0.039 | -0.174 |
| 96 | 70 | 0.000 | -0.063 | -0.028 | -0.024 | -0.040 | -0.016 | -0.072 |
| 96 | 80 | 0.000 | 0.027 | 0.012 | 0.010 | 0.017 | 0.007 | 0.031 |
| 96 | 90 | 0.000 | 0.117 | 0.051 | 0.045 | 0.075 | 0.030 | 0.133 |
| 96 | 100 | 0.000 | 0.208 | 0.091 | 0.080 | 0.133 | 0.053 | 0.235 |
| 0 | 77 | -0.675 | -0.592 | -0.273 | -0.199 | -0.332 | -0.243 | -0.525 |
| 10 | 77 | -0.605 | -0.530 | -0.244 | -0.178 | -0.297 | -0.218 | -0.471 |
| 20 | 77 | -0.535 | -0.469 | -0.216 | -0.157 | -0.263 | -0.192 | -0.416 |
| 30 | 77 | -0.464 | -0.407 | -0.188 | -0.137 | -0.228 | -0.167 | -0.361 |
| 40 | 77 | -0.394 | -0.345 | -0.159 | -0.116 | -0.194 | -0.142 | -0.306 |
| 50 | 77 | -0.324 | -0.284 | -0.131 | -0.095 | -0.159 | -0.116 | -0.252 |
| 60 | 77 | -0.253 | -0.222 | -0.102 | -0.074 | -0.125 | -0.091 | -0.197 |
| 70 | 77 | -0.183 | -0.160 | -0.074 | -0.054 | -0.090 | -0.066 | -0.142 |
| 80 | 77 | -0.113 | -0.099 | -0.045 | -0.033 | -0.055 | -0.041 | -0.088 |
| 90 | 77 | -0.042 | -0.037 | -0.017 | -0.012 | -0.021 | -0.015 | -0.033 |
| 100 | 77 | 0.028 | 0.025 | 0.011 | 0.008 | 0.014 | 0.010 | 0.022 |

## Scenario analyses

**Figure D1a: Maximum price at which the biomarker test is cost-effective at each sensitivity (91-100%) and specificity (0-100%) pair for the biomarker test vs. standard test pathway** **of TAB and clinical judgment (starting age of 51)**

**
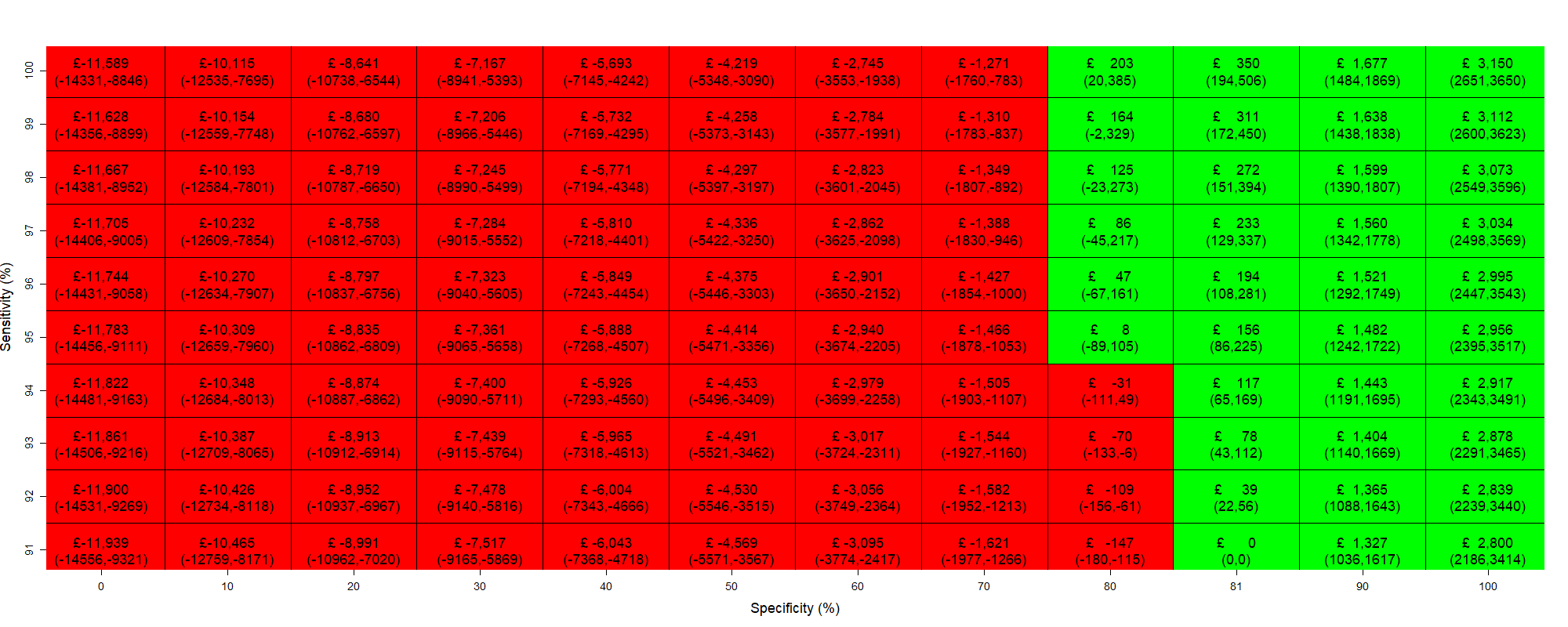
**

**Figure D1b: Maximum price at which the biomarker test is cost-effective at each sensitivity (0-100%) and specificity (81-99%) pair for the biomarker test vs. standard test pathway** **of TAB and clinical judgment (starting age of 51)**

**
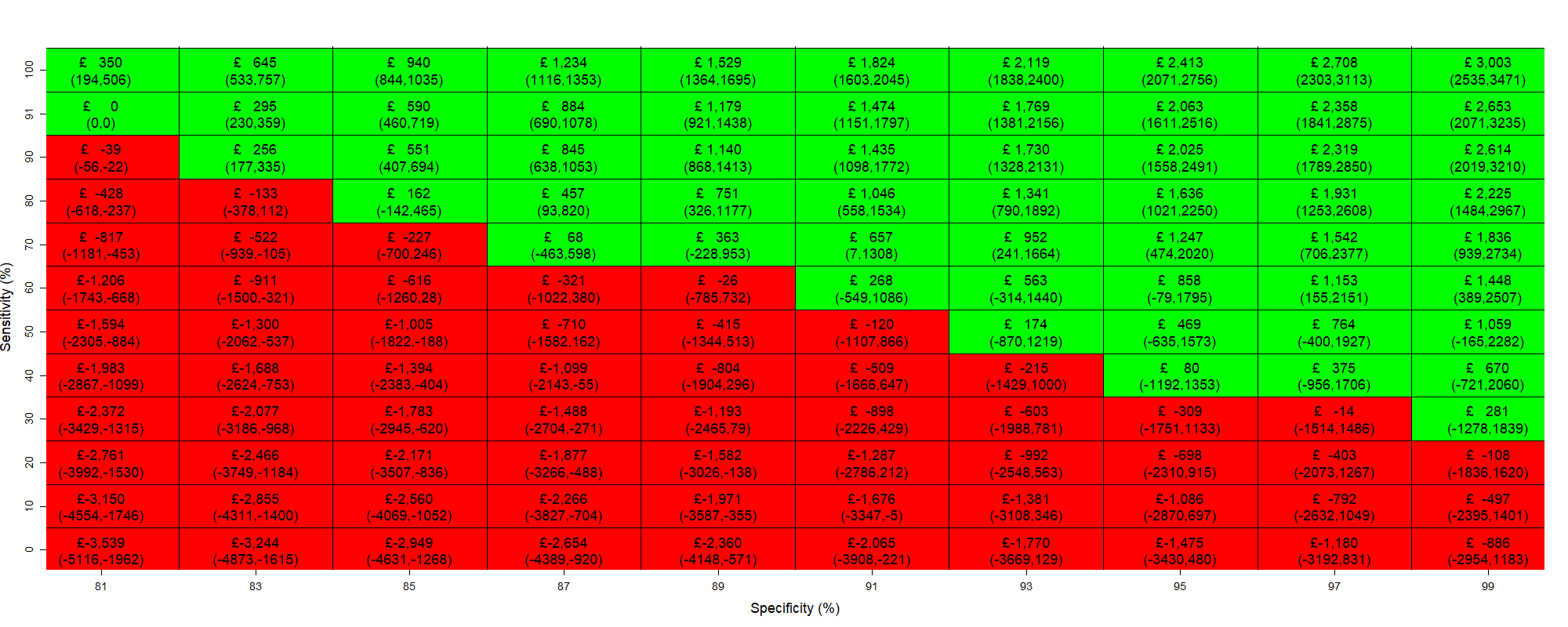
**

**Figure D2a: Maximum price at which the biomarker test is cost-effective at each sensitivity (91-100%) and specificity (0-100%) pair for the biomarker test vs. standard test pathway** **of TAB and clinical judgment (50% TNs treated as TPs)**

**
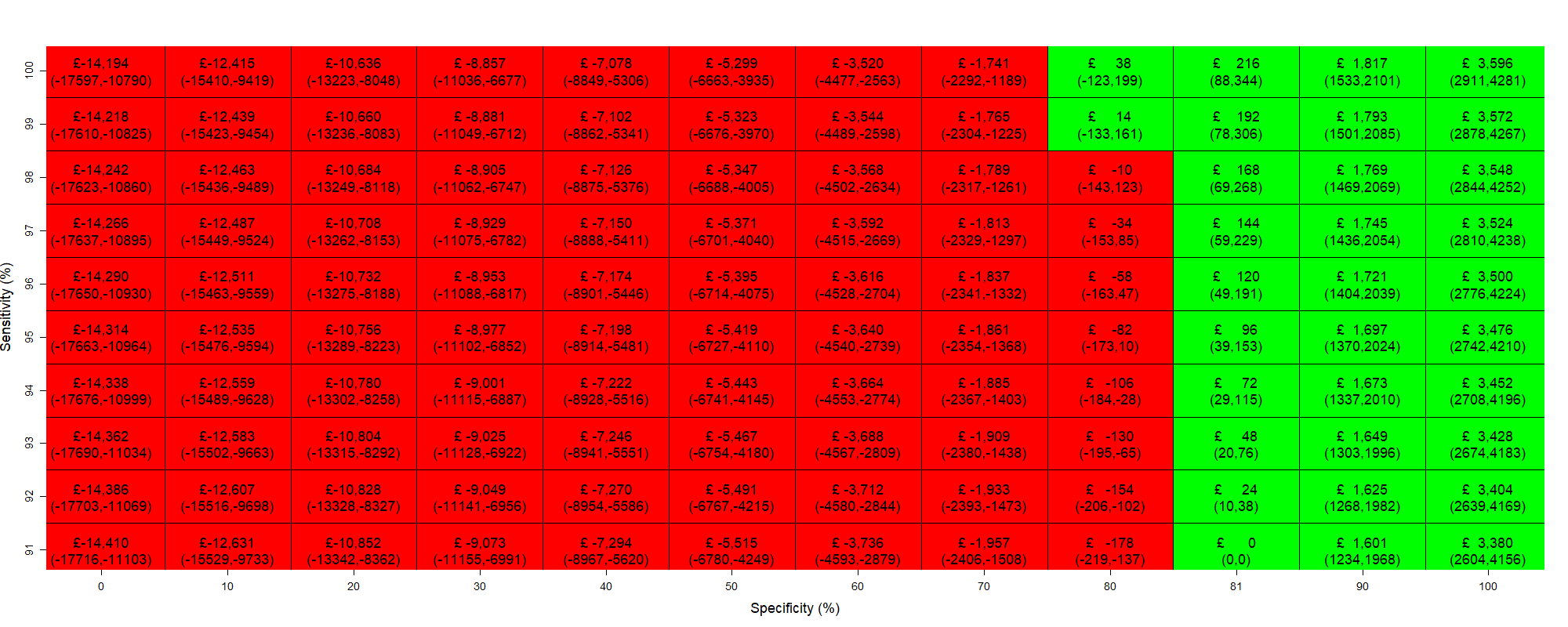
**

**Figure D2b: Maximum price at which the biomarker test is cost-effective at each sensitivity (0-100%) and specificity (81-99%) pair for the biomarker test vs. standard test pathway** **of TAB and clinical judgment (50% TNs treated as TPs)**

**
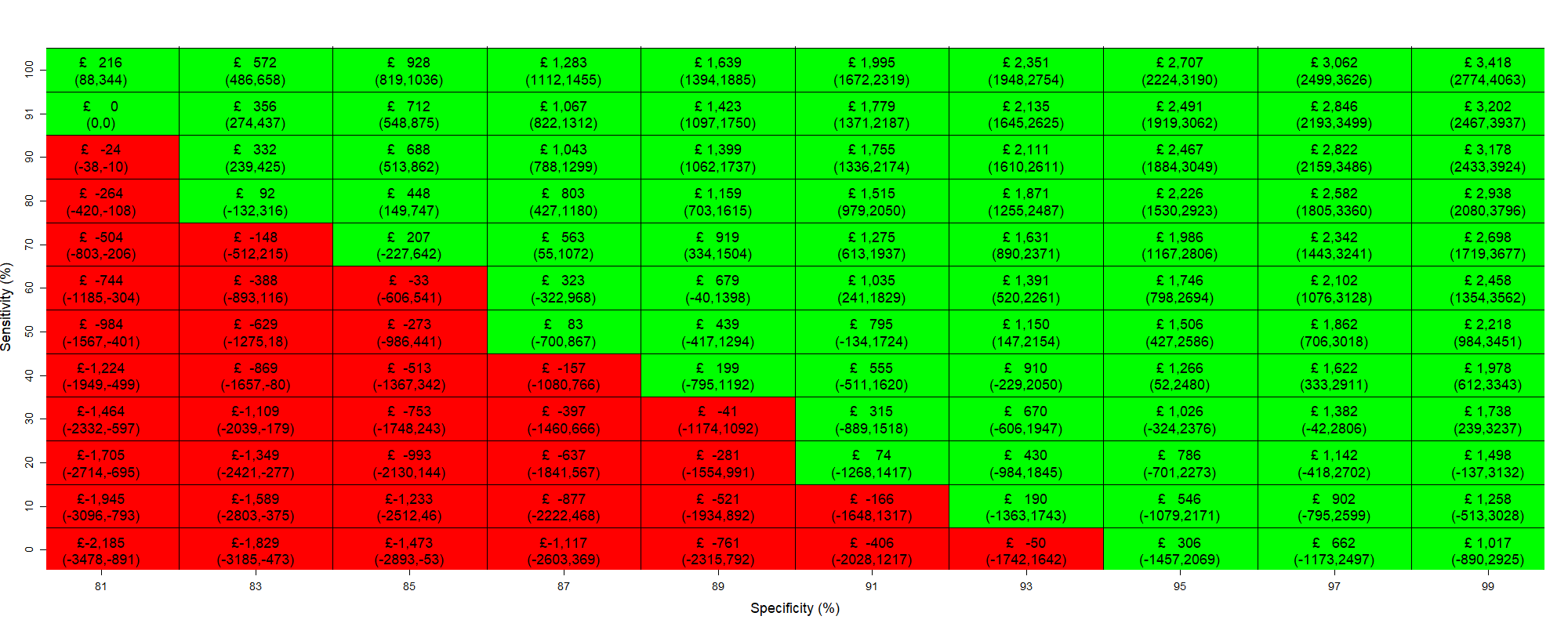
**

**Figure D3a: Maximum price at which the biomarker test is cost-effective at each sensitivity (91-100%) and specificity (0-100%) pair for the biomarker test vs. standard test pathway** **of TAB and clinical judgment (75% TNs treated as TPs)**

**
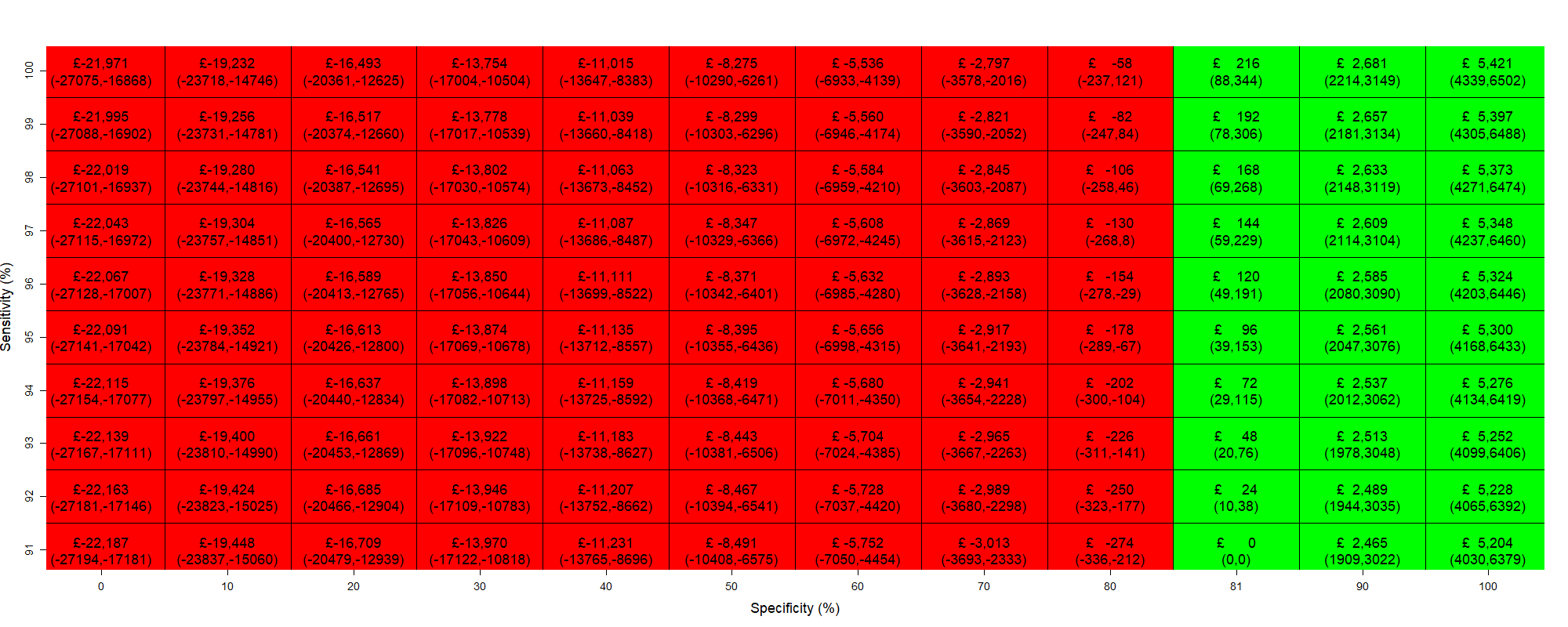
**

**Figure D3b: Maximum price at which the biomarker test is cost-effective at each sensitivity (0-100%) and specificity (81-99%) pair for the biomarker test vs. standard test pathway** **of TAB and clinical judgment (75% TNs treated as TPs)**

**
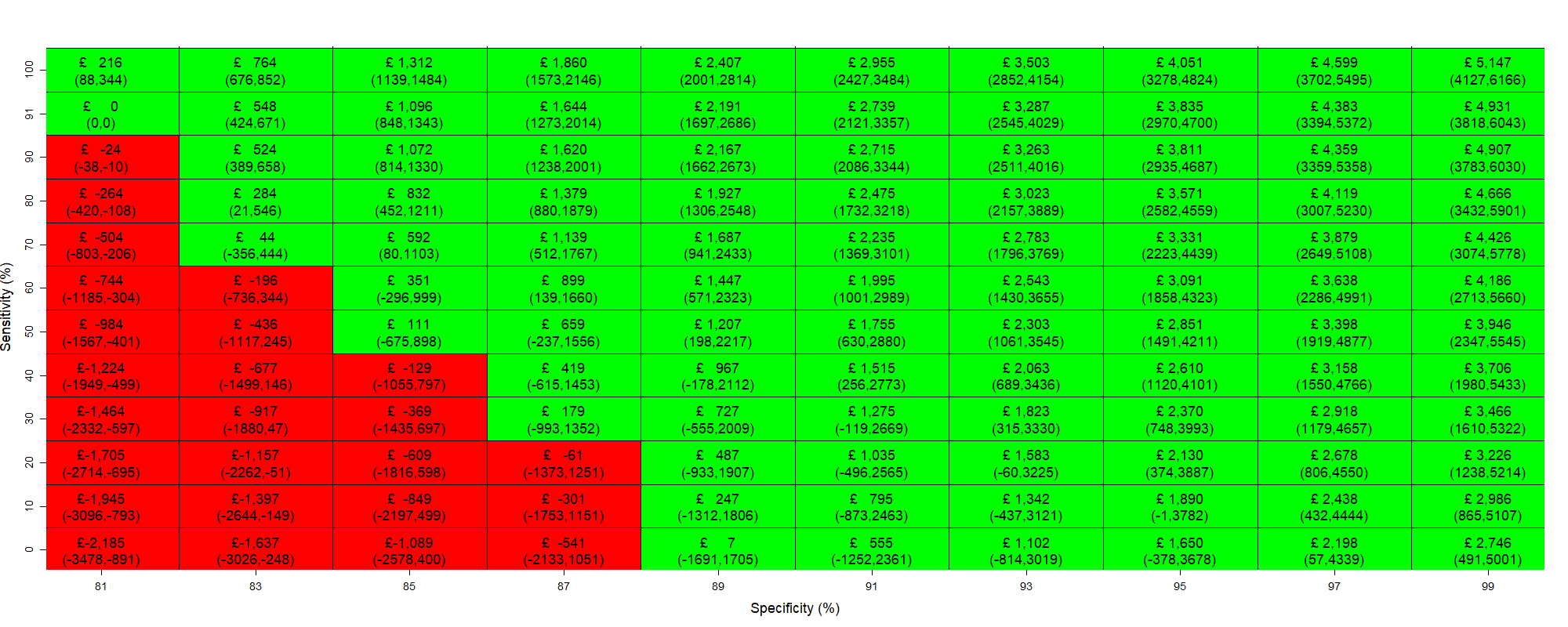
**

**Figure D4a: Maximum price at which the biomarker test is cost-effective at each sensitivity (91-100%) and specificity (0-100%) pair for the biomarker test vs. standard test pathway** **of TAB and clinical judgment (20-year time horizon)**


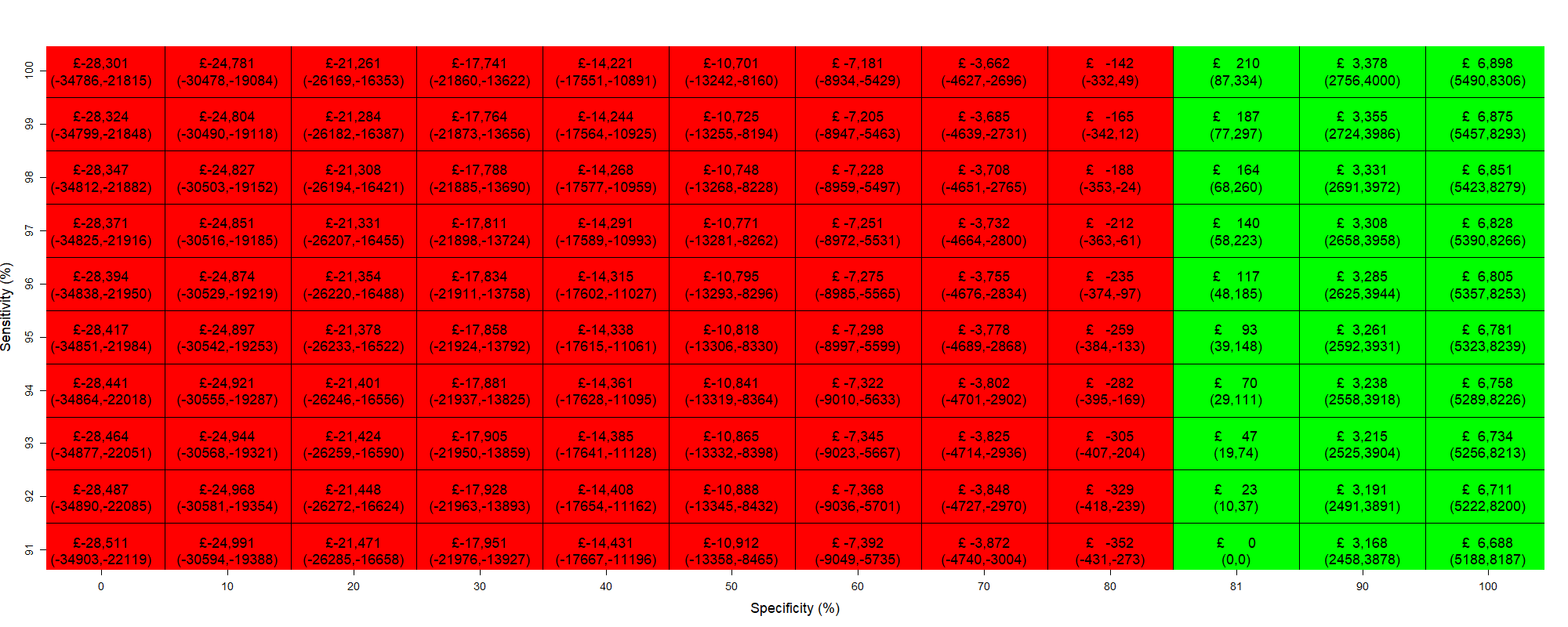


**Figure D4b: Maximum price at which the biomarker test is cost-effective at each sensitivity (0-100%) and specificity (81-99%) pair for the biomarker test vs. standard test pathway** **of TAB and clinical judgment (20-year time horizon)**


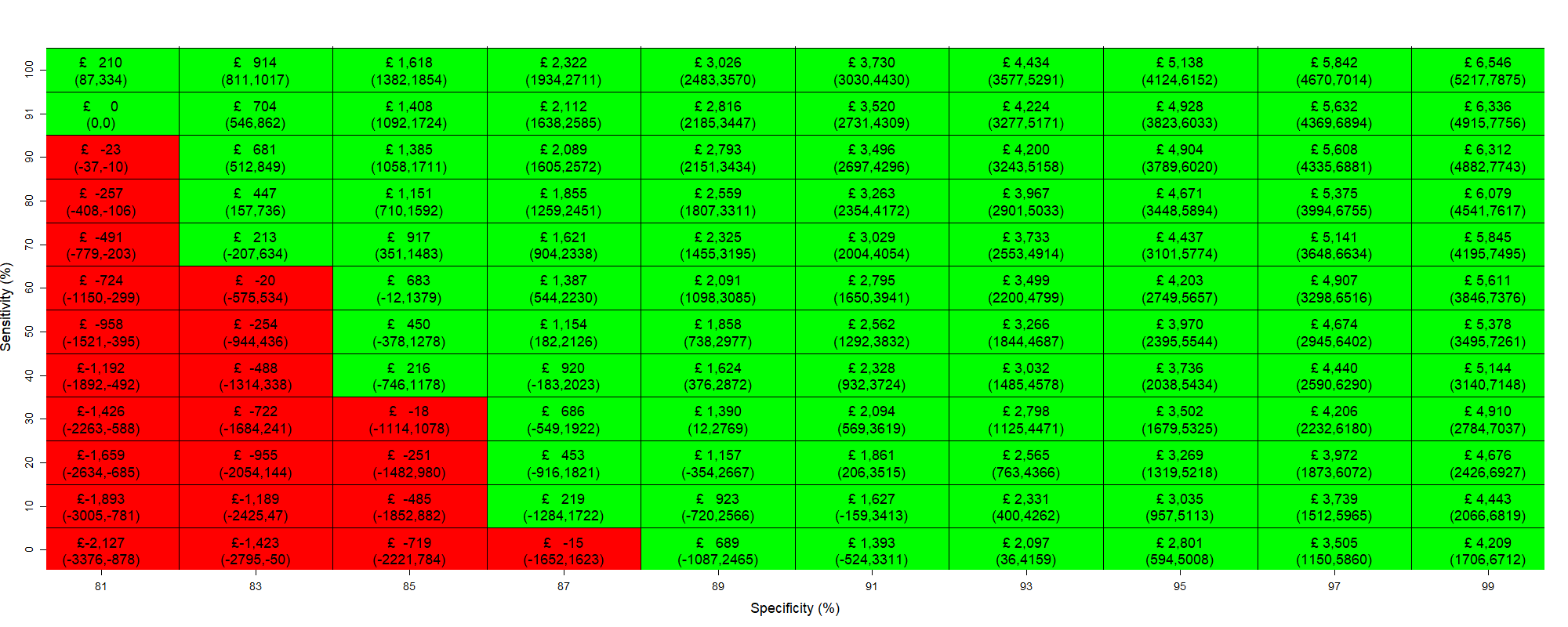


**Figure D5a: Maximum price at which the biomarker test is cost-effective at each sensitivity (91-100%) and specificity (0-100%) pair for the biomarker test vs. standard test pathway** **of TAB and clinical judgment (10-year time horizon)**


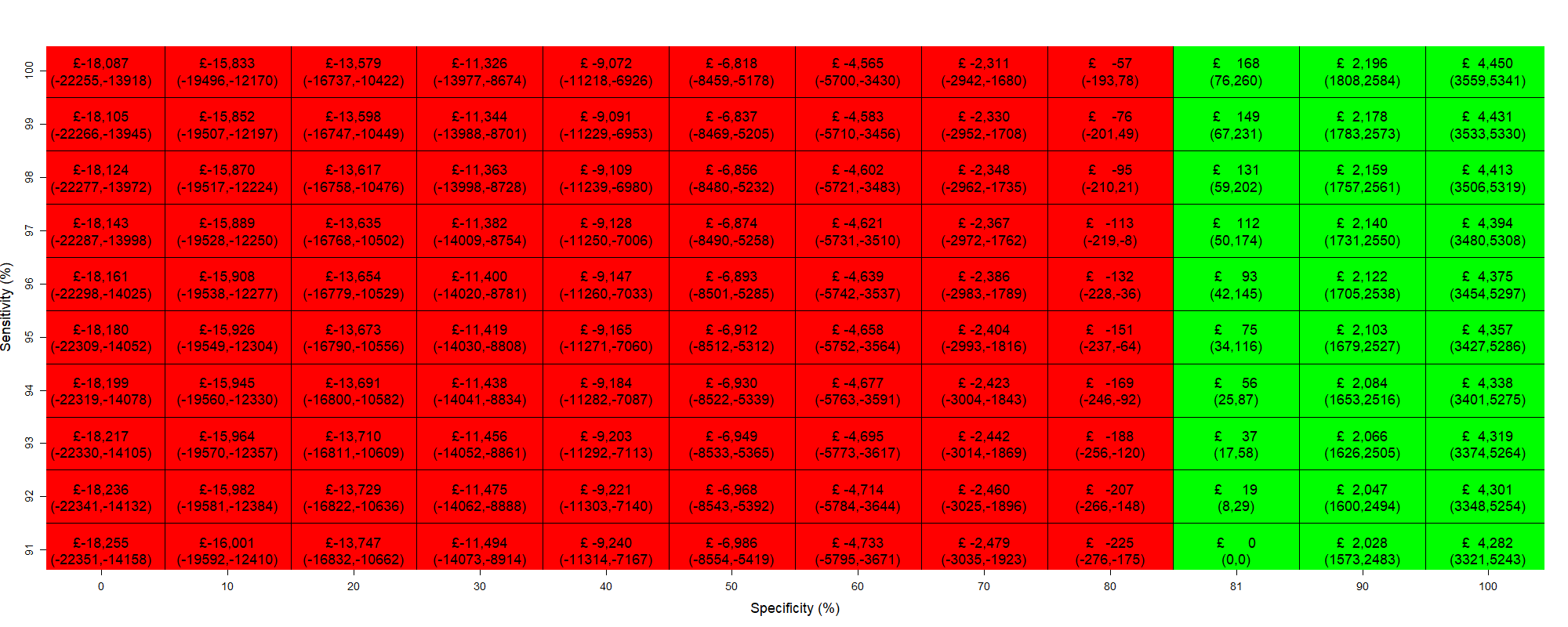


**Figure D5b: Maximum price at which the biomarker test is cost-effective at each sensitivity (0-100%) and specificity (81-99%) pair for the biomarker test vs. standard test pathway** **of TAB and clinical judgment (10-year time horizon)**


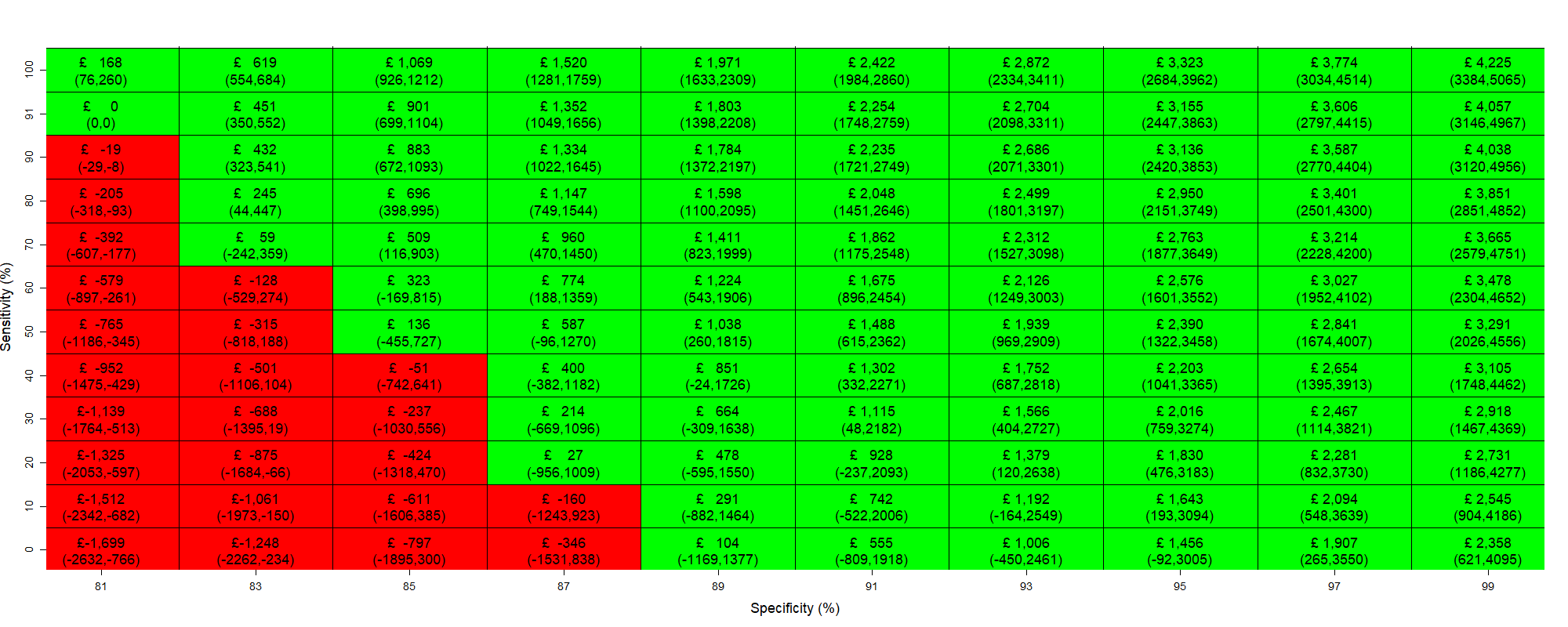


**Figure D6a: Maximum price at which the biomarker test is cost-effective at each sensitivity (91-100%) and specificity (0-100%) pair for the biomarker test vs. standard test pathway** **of TAB and clinical judgment (5-year time horizon)**


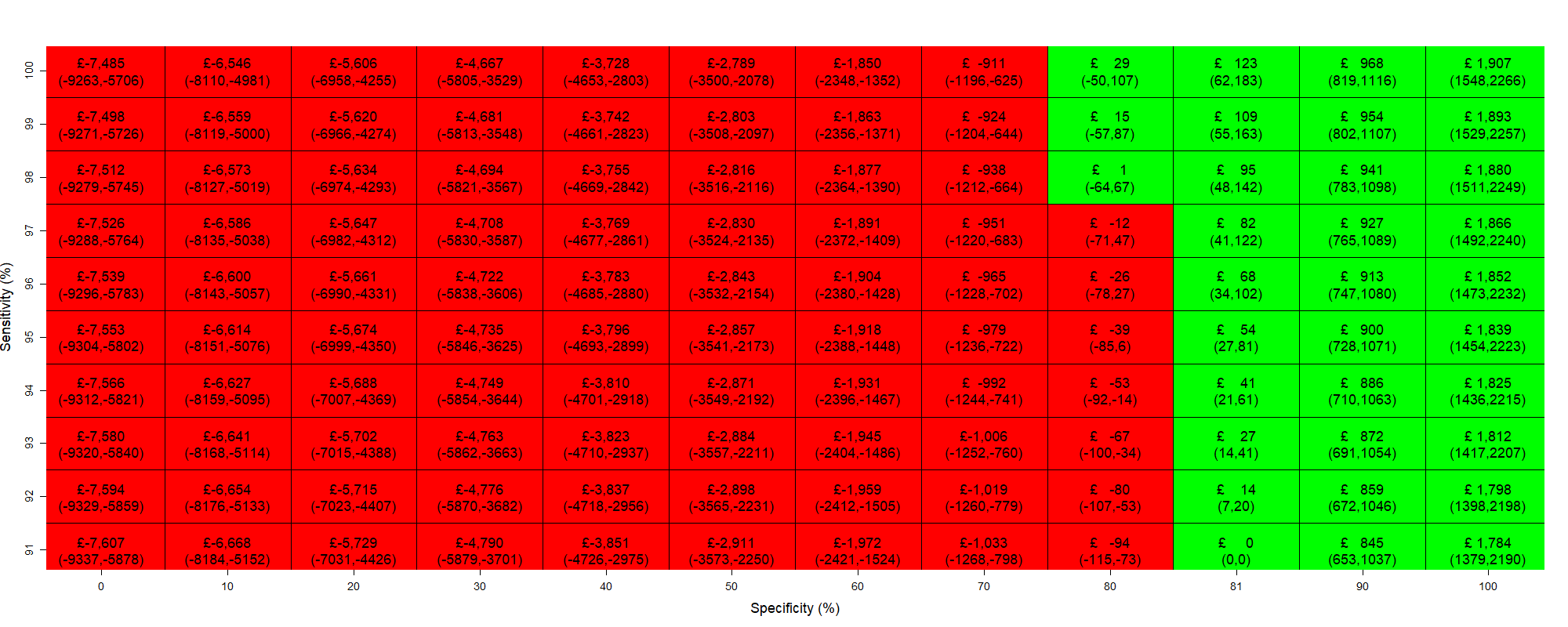


**Figure D6b: Maximum price at which the biomarker test is cost-effective at each sensitivity (0-100%) and specificity (81-99%) pair for the biomarker test vs. standard test pathway** **of TAB and clinical judgment (5-year time horizon)**


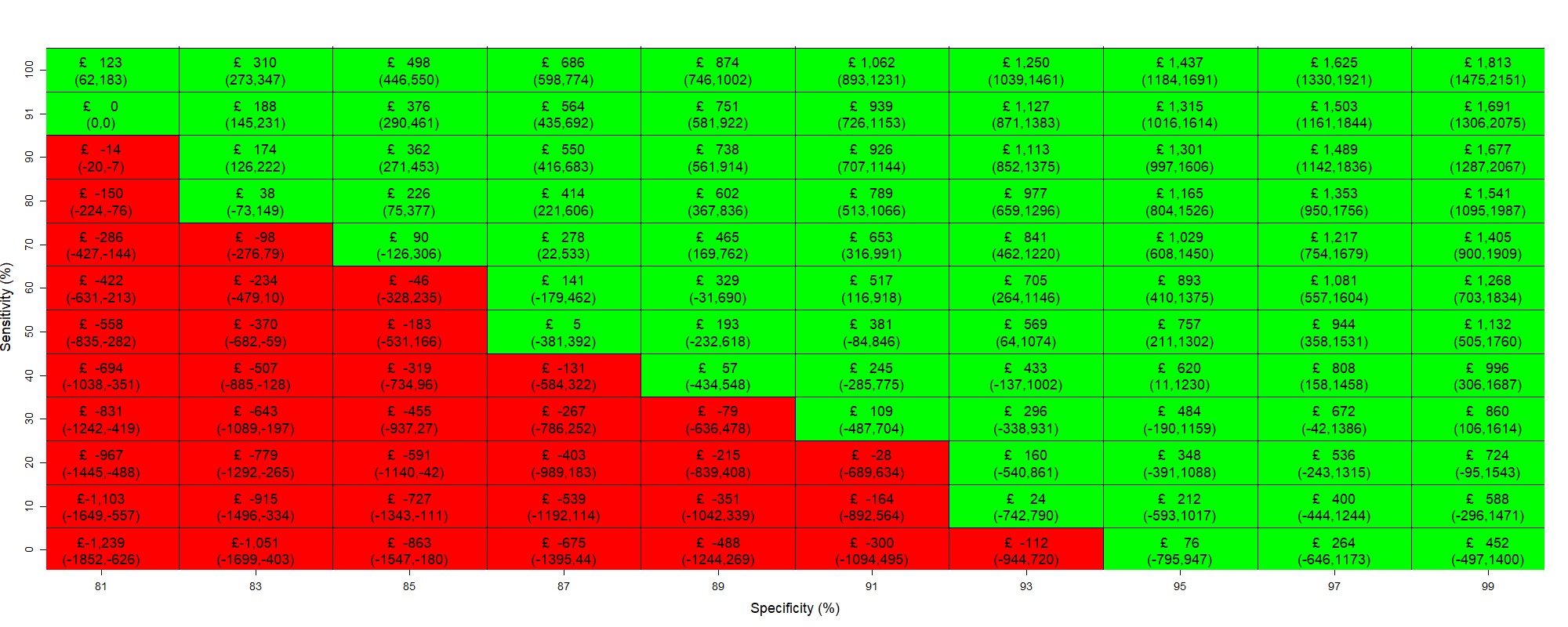


**Figure D7a: Maximum price at which the biomarker test is cost-effective at each sensitivity (91-100%) and specificity (0-100%) pair for the biomarker test vs. standard test pathway** **of TAB and clinical judgment (2-year glucocorticoid duration)**


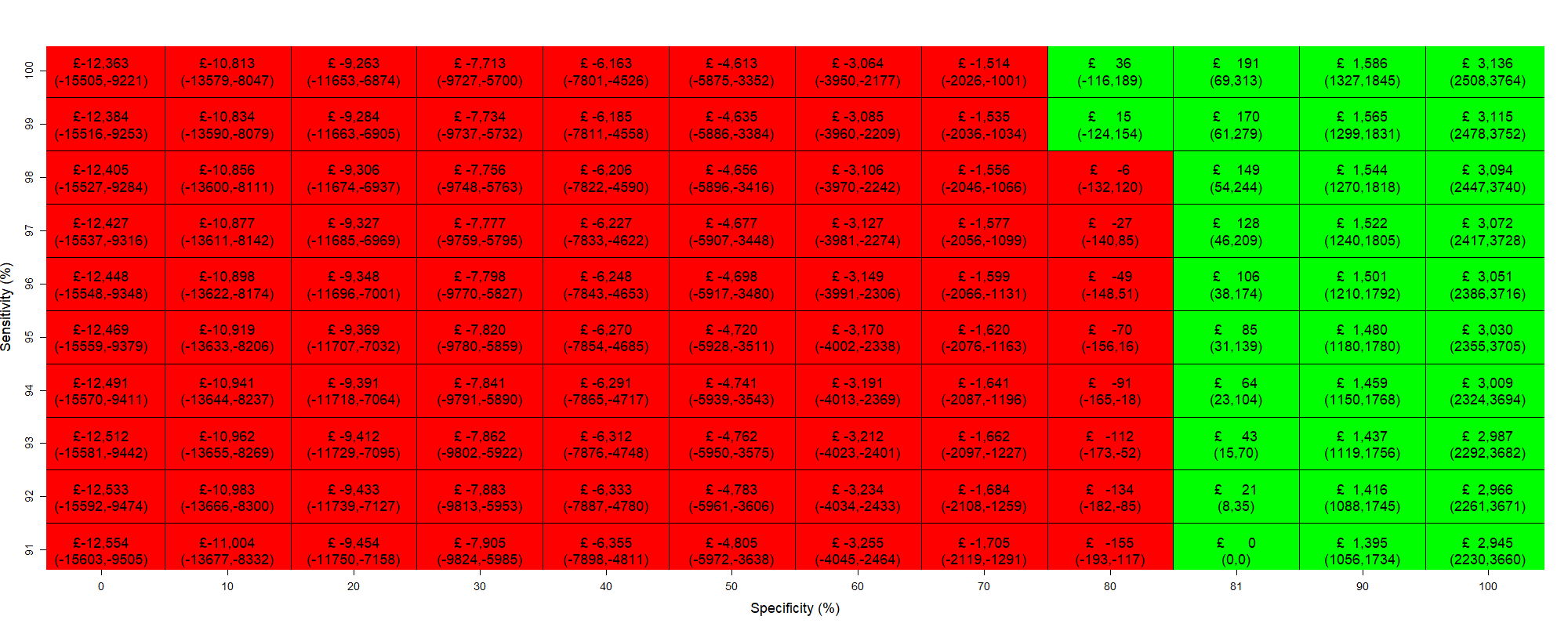


**Figure D7b: Maximum price at which the biomarker test is cost-effective at each sensitivity (0-100%) and specificity (81-99%) pair for the biomarker test vs. standard test pathway** **of TAB and clinical judgment (2-year glucocorticoid duration)**


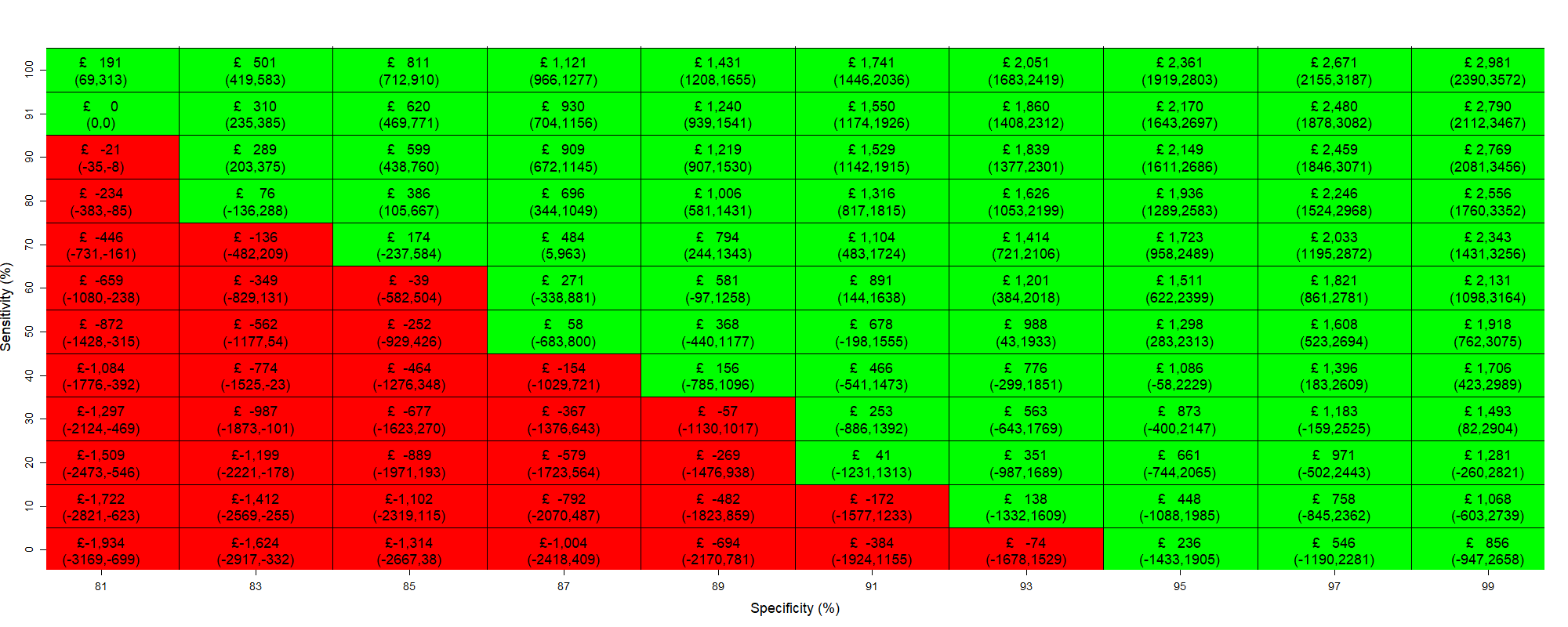


**Figure D8a: Maximum price at which the biomarker test is cost-effective at each sensitivity (91-100%) and specificity (0-100%) pair for the biomarker test vs. standard test pathway** **of TAB and clinical judgment (10-year glucocorticoid duration)**


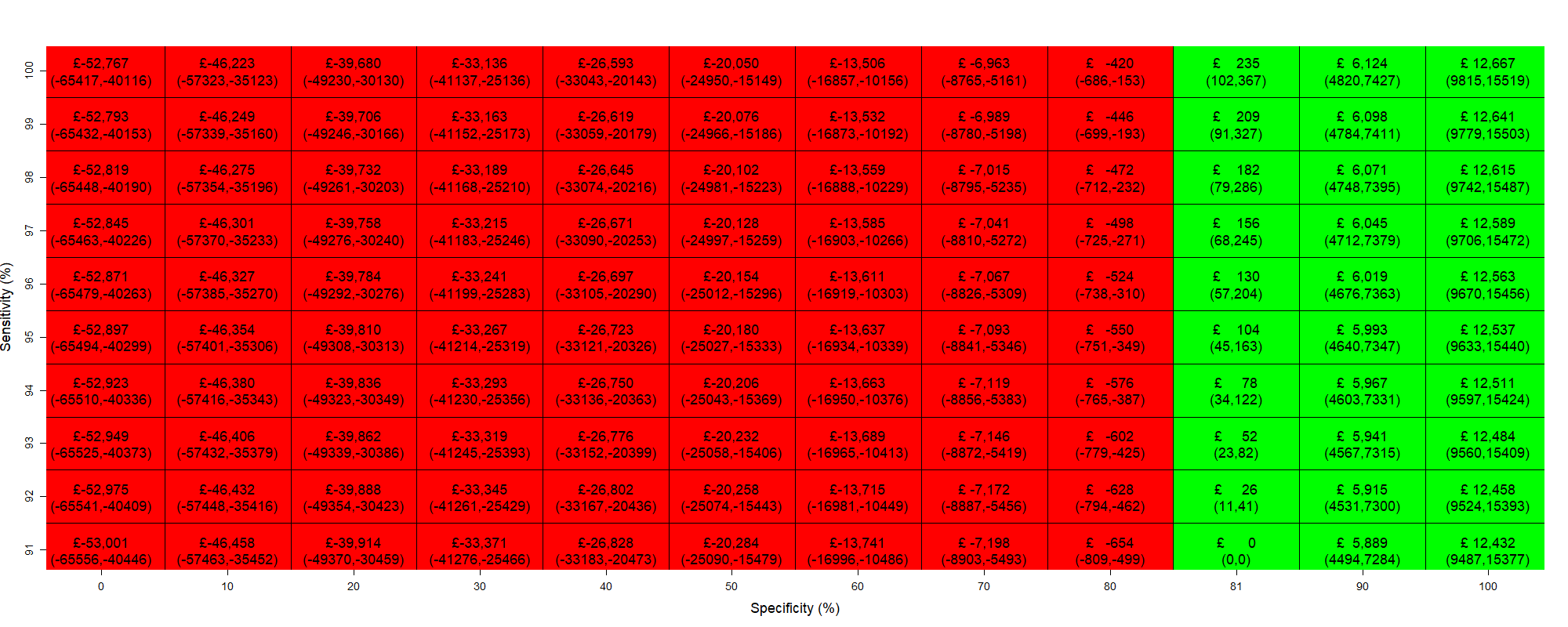


**Figure D8b: Maximum price at which the biomarker test is cost-effective at each sensitivity (0-100%) and specificity (81-99%) pair for the biomarker test vs. standard test pathway** **of TAB and clinical judgment (10-year glucocorticoid duration)**


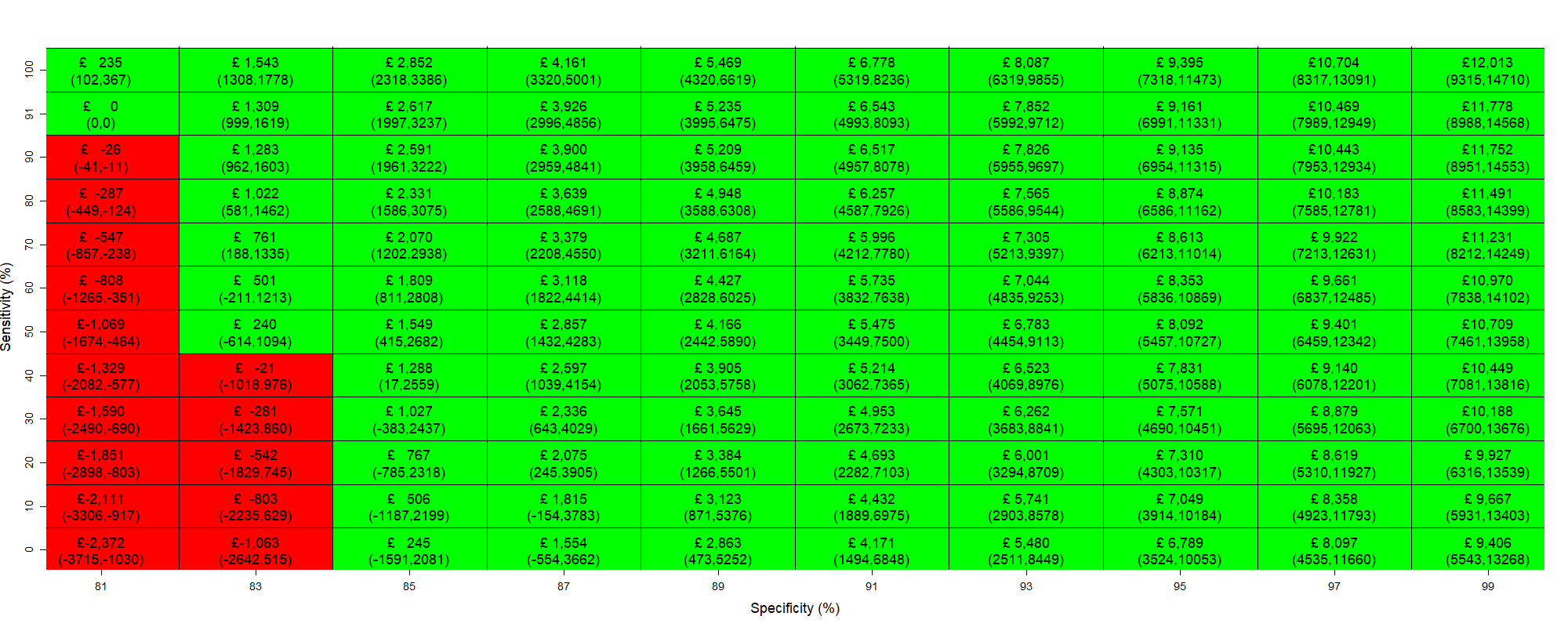

Supplement: online supplemental file 1 [file bmjopen-15-11-s001.docx]
