# Supplementary material for: Early-stage cost-utility analysis of novel diagnostic tests for giant cell arteritis: a modelling study in UK secondary care
Source: BMJ Open. 2025 Nov 13;15(11):e102888. doi: 10.1136/bmjopen-2025-102888 (PMC12625940; doi:10.1136/bmjopen-2025-102888)
Supplement: Supplementary file 2 [file bmjopen-2025-102888.reviewer_comments.pdf]

## PEER REVIEW HISTORY

BMJ Open publishes all reviews undertaken for accepted manuscripts. Reviewers are asked to complete a checklist review form and are provided with free text boxes to elaborate on their assessment. These free text comments are reproduced below.

### ARTICLE DETAILS

**Title (Provisional)**

Early-stage cost-utility analysis of novel diagnostic tests for giant cell arteritis: a modelling study in UK secondary care

**Authors**

Yang, Miaoqing; Cocco, Paola; Mackie, Sarah; Morgan, Ann

---

### VERSION 1 - REVIEW

---

| | |
|---|---|
| **Reviewer** | **1** |
| **Name** | **Green, Kile** |
| **Affiliation** | **Newcastle University, NIHR In Vitro Diagnostics Co-operative** |
| **Date** | **04-Jul-2025** |
| **COI** | **None** |

---

Overall well considered and thorough paper, well done to the research team. Thank you for including the CHEERS checklist in the submission. Some minor typos and grammatical errors should be corrected in revision.

This article describes a headroom analysis for hypothetical biomarker tests designed as add-ons to current care. The article outlines scenarios and conditions in which a novel test of appropriate cost, sensitivity, and specificity may add value to the system. A nicely designed, and straight-forward model was developed which may offer value and reference to diagnostic developers working in this space.

The abstract is neat and succinct. In the abstract results section, a more interesting headline might focus on what a hypothetical developer will need to achieve to add value to the system, describing which parameters offer the greatest value (specificity increase seems more influential than sensitivity, lines 3-20, page 13) might be more useful to a reader than the headroom for a perfect test. I understand word limits may restrict what you can add here.

The introduction is clear and well-pitched for a general scientific audience covering the condition in question, simple narrative diagnostic pathway, and a brief introduction to the purpose of the study and the methods employed. No concerns here.

Methods are appropriate and well described. Page 5 line 60 notes author initials without context, perhaps just adding 'consensus amongst clinical experts AWM and SLM'. Section 2.2 could include reference to R packages used to build the model if any were used. 2.3.1 seems reasonable - a single point of entry for the hypothetical tests was modelled, and appears to be a reasonable position for such a biomarker test (as an add-on to existing standard of care, prior to confirmatory testing).

Clear listing of model assumptions in 2.5(1st), thank you.

Note - section numbers between 2.3 and 2.6 are out of order and should be corrected before publication. There are two 2.5 sections, one of which comes prior to 2.4. Error does not affect the content or meaning.

I assume data transfer agreements already exist for any data transferred between Leeds Teaching Hospitals NHS Trust and the research team given their close association, depending on exactly what content was shared in relation to 2.4.1 - may be worth describing in an ethics and consent section.

Possibly a typo in 2.4.3, page 9, line 27-30, sentence is difficult to read, but can be understood.

2.5(2nd) 'Analysis and dealing with uncertainty' section is well written, reasonable analysis.

2.6 is slightly difficult to comment on as it is unclear if the clinical experts are part of the research team or independent. It is possible the clinical advisors were also acting from the perspective of patient advocates, which may constitute PPIE. The consultation work with other clinicians may be classified as research, rather than involvement or engagement. The authors do note involvement in 'interpretation and presentation', which would be considered an appropriate PPIE activity. For a relatively self-contained, and largely quantitative project such as this, meaningful PPIE may be difficult to achieve, but authors could consider patient co-design or patient/public involvement in an expert advisory group to steer the research or inform dissemination strategies in the future. For this study, additional PPIE support to develop a lay summary, social media post, or other dissemination activity may be a suitable engagement activity at this stage and add public interest value to the research. Regardless, it appears the authors have considered the patient perspective in their work.

Section 3 is well written and informative, suitably addressing the research question. Tables and figures are appropriate, referenced correctly throughout, and utilised well to describe and convey the content. Supplementary materials appear complete. Thank you.

Section 4 is useful and explores the major findings of the study. The discussion is appropriate and aligns with the results. It could have discussed the clinical impact of introducing a novel

test (minor, but positive improvement of health outcomes such as stoke and diabetes), but these were minor findings compared to the headroom analysis and aren't necessary for inclusion. Perhaps remove the ':' symbol from the title of 4.1.

Good implications and future thinking, there are perhaps some caveats on where and how a test will be used in the care pathway that might impact this headroom - replacement test rather than add-on, earlier or later testing, intended population and inclusion criteria etc, but within a typical biomarker implementation pathway this framework provides a good overview of the general headroom available for novel diagnostics.

Input parameter table is complete and well referenced.

Overall a well written paper, some minor typos, but factually complete. Nice, simple, uncomplicated decision model and clear transition model. Reasonable discussion based on the results identified. This article would add value for researchers, healthcare professionals and industry partners involved in biomarker development in giant cell arteritis.

---

| | |
|---|---|
| **Reviewer** | **2** |
| **Name** | **Girdwood, Sarah** |
| **Affiliation** | **FIND, Impact** |
| **Date** | **18-Jul-2025** |
| **COI** | **None** |

---

Summary

This study presents an early-stage cost-utility analysis to evaluate the potential value of hypothetical diagnostic biomarker tests for Giant Cell Arteritis (GCA). Using a combined decision-tree and Markov modeling approach, the analysis estimates ICERs and maximum cost-effective prices across varying sensitivity and specificity levels, compared to current diagnostic pathways. Results indicate that a rule-out test (confirmatory test) with high specificity to reduce false positives and thereby limit unnecessary glucocorticoid exposure is particularly valuable given the toxicity of continued empiric treatment.

• Section 2.1: In the Methods section, the authors refer to conducting a “targeted systematic review of economic literature” and a “targeted systematic search.” This terminology is misleading, as no systematic review was conducted. The TABUL study plays a central role in shaping the model structure and parameter selection in this analysis. Given its influence, it would be more appropriate to introduce and contextualize the TABUL study earlier, ideally in the Introduction, rather than presenting it only in the Methods. Section 2.1 might be better titled “Model design and structure”, with a clear explanation of how the model was adapted or extended from the TABUL framework, and which parameters were drawn directly from it.

o Additionally, the rationale for limiting the search to studies with “economic evaluation” keywords is unclear. Many key parameters for the model are not economic in nature so this restriction may have led to the exclusion of relevant studies. Similarly, the decision to restrict the literature to UK-based studies is somewhat limiting. While UK data are appropriate for costing and population-level inputs, diagnostic performance data from non-UK settings could still provide valuable insights, particularly where UK data are limited or outdated.

o The following sentence doesn’t feel necessary, nor do the authors need to justify it. “Due to resource limitations, we focused on identifying appropriate data sources rather than conducting a meta-analysis for each input parameter.” Rather perhaps authors can just say they prioritised the use of published literature and expert opinion over formal meta-analyses.

• Please clarify why a 19-month glucocorticoid duration was assumed. Indicate whether this reflects guidelines or typical prescribing practice, and note that glucocorticoids are not usually used long-term for GCA.

o Also, consider commenting on how long AE risks persist after stopping treatment and whether these risks are assumed to decline over time in the model.

• Clarify terminology for international readers—for example, briefly explain abbreviations for PSSRU and briefly describe what the PSS perspective includes.

• Consider using a starting age of 50, as GCA typically affects individuals aged 50 and older. While 71 reflects the TABUL cohort average, modeling from age 50 may better align with clinical onset and would likely increase headroom for cost-effectiveness.

• Given the higher prevalence of GCA in women (e.g. 72% of study cohort is women in TABUL) and gender differences in life expectancy and risk profiles, this seems like a limitation to the study which could meaningfully affect results. The authors should either justify this approach more clearly or consider conducting a gender-stratified or weighted analysis, or at minimum, include a sensitivity analysis with female values for prevalence, life expectancy etc to assess its impact.

• Performance

o Explicitly state that the model assumes a combined diagnostic accuracy based on biomarker tests plus existing blood tests (e.g. ESR, CRP), reflecting overall clinical decision-making. This should be stated explicitly, so manufacturers understand the performance target applies to the full algorithm, not the biomarker test alone. Otherwise, there’s a risk of misinterpreting the required sensitivity and specificity, which may not be achievable by the biomarker in isolation.

o In the TAB + US+ clinical judgement algorithm, wasn’t TAB only conducted if US was positive? How was this sequential testing treated in the model?

• Consider relaxing the assumption of full clinician adherence to test results and treatment guidelines. In practice, clinicians may continue or stop glucocorticoids based on clinical judgment, even with negative/positive test results (Doctor does not test – uses clinical judgement). If no data are available, consider reducing adherence from 100% to a more realistic estimate in sensitivity analysis and show how it affects the price of the new hypothetical test.

• Also, would a less invasive, faster test increase testing access. E.g. not everyone gets a TAB now, but if it is a non- or less invasive biomarker test, would confirmatory testing increase thereby increasing access and reducing empiric treatment duration?

o On a related note regarding the timing of the new diagnostic test: the current algorithm is within 2 weeks, how would an instant biomarker test improve outcomes? How long does this new algorithm with blood tests take? If shorter, does the model incorporate these benefits (namely, reduced empiric treatment duration)?

• Is there any reason to believe that GCA glucocorticoid AEs are at a lower rate to non-GCA glucocorticoid AEs.

• Consider using "maximum cost-effective price" or define "headroom" on first use, as it may not be familiar to all readers.

• Consider including a cost-neutral price—the price at which the new test yields the same cost/QALY outcomes as standard care. This may be more realistic for reimbursement (tempering manufacturing expectations) and more useful for policymakers and manufacturers. This may be closer to the 22 pound biomarker test.

• In the Discussion, it would be useful to have more of a discussion contextualising these results with regards to the TABUL study. How does this study add value to the TABUL study. How does it differ? Etc.

• Figure 2a and 2b have the same figure caption. Similarly, figure 3a and 3b. Add more detail around the range of sensitivity and specificity used so that they can be understood in isolation.

• The purpose of Figures 3a and 3b is unclear. While they illustrate the QALY gains across varying test accuracy, these results are difficult to interpret without knowing what biomarker test price is assumed? If the goal is to show how small a QALY gain can still be cost-effective, this would need to be paired with the cost assumptions. Otherwise, Figures 2a/2b (headroom price) already capture the actionable trade-off between performance and price. Please consider clarifying the intent of these figures, as well as the biomarker price or removing them if they do not add interpretive value.

• Results: Would consider colour coding positive and negatives to make Table 2 easier to read. Are the numbers for vision loss across specificity correct (all zero)?

• Results: There is no mention of the PSA? Was this conducted?

## VERSION 1 - AUTHOR RESPONSE

**Reviewer: 1**
**Dr. Kile Green, Newcastle University, Newcastle University**
**Comments to the Author:**
Overall well considered and thorough paper, well done to the research team. Thank you for including the CHEERS checklist in the submission. Some minor typos and grammatical errors should be corrected in revision. This article describes a headroom analysis for hypothetical biomarker tests designed as add-ons to current care. The article outlines scenarios and conditions in which a novel test of appropriate cost, sensitivity, and specificity may add value to the system. A nicely designed, and straight-forward model was developed which may offer value and reference to diagnostic developers working in this space.

*Thank you very much for your kind feedback. We have carefully reviewed the manuscript again and corrected typos and grammatical errors.*

The abstract is neat and succinct. In the abstract results section, a more interesting headline might focus on what a hypothetical developer will need to achieve to add value to the system, describing which parameters offer the greatest value (specificity increase seems more influential than sensitivity, lines 3-20, page 13) might be more useful to a reader than the headroom for a perfect test. I understand word limits may restrict what you can add here.

*Thank you. We have highlighted in the abstract that improvements in test specificity have a greater impact on cost-effectiveness than sensitivity.*

The introduction is clear and well-pitched for a general scientific audience covering the condition in question, simple narrative diagnostic pathway, and a brief introduction to the purpose of the study and the methods employed. No concerns here. *Thank you for your kind comment.*
Methods are appropriate and well described. Page 5 line 60 notes author initials without context, perhaps just adding 'consensus amongst clinical experts AWM and SLM'.

*Thank you. Changes have been made.*

Section 2.2 could include reference to R packages used to build the model if any were used. *Thank you. The decision tree model was built from scratch using base R functions and general-purpose packages (e.g., dplyr, ggplot2) rather than a dedicated health economic modelling package. Our modelling approach follows the principles of the DARTH framework for decision-analytic modelling in R (https://github.com/DARTH-git). We have now added this information to the Methods section to improve transparency*

*and reproducibility.*
2.3.1 seems reasonable - a single point of entry for the hypothetical tests was modelled, and appears to be a reasonable position for such a biomarker test (as an add-on to existing standard of care, prior to confirmatory testing). *Thank you for your kind comment.*
Clear listing of model assumptions in 2.5(1st), thank you. *Thank you for your kind comment.*
Note - section numbers between 2.3 and 2.6 are out of order and should be corrected before publication. There are two 2.5 sections, one of which comes prior to 2.4. Error does not affect the content or meaning. *Thank you for pointing out the error. All section numbers have been corrected.*
I assume data transfer agreements already exist for any data transferred between Leeds Teaching Hospitals NHS Trust and the research team given their close association, depending on exactly what content was shared in relation to 2.4.1 - may be worth describing in an ethics and consent section. *Thank you. A data transfer agreement was not required, as the input parameter on baseline GCA prevalence was derived from a published figure based on local data from Leeds Teaching Hospitals NHS Trust.*
Possibly a typo in 2.4.3, page 9, line 27-30, sentence is difficult to read, but can be understood. *Thank you. The sentence has been revised.*
2.5(2nd) 'Analysis and dealing with uncertainty' section is well written, reasonable analysis. *Thank you for your kind comment.*
2.6 is slightly difficult to comment on as it is unclear if the clinical experts are part of the research team or independent. It is possible the clinical advisors were also acting from the perspective of patient advocates, which may constitute PPIE. The consultation work with other clinicians may be classified as research, rather than involvement or engagement. The authors do note involvement in 'interpretation and presentation', which would be considered an appropriate PPIE activity. For a relatively self-contained, and largely quantitative project such as this, meaningful PPIE may be difficult to achieve, but authors could consider patient co-design or patient/public involvement in an expert advisory group to steer the research or inform dissemination strategies in the future. For this study, additional PPIE support to develop a lay summary, social media post, or other dissemination activity may be a suitable engagement activity at this stage and add public interest value to the research. Regardless, it appears the authors have considered the patient perspective in their work. *Thank you for your comment. We held a stakeholder workshop at the outset of this project to explore unmet clinical needs in GCA diagnosis. This involved clinical experts, industry partners, and a patient representative. We have now added relevant details to the PPIE section to clarify the stakeholder involvement.*

Section 3 is well written and informative, suitably addressing the research question. Tables and figures are appropriate, referenced correctly throughout, and utilised well to describe and convey the content. Supplementary materials appear complete. Thank

you. *Thank you for your kind comment.*
Section 4 is useful and explores the major findings of the study. The discussion is appropriate and aligns with the results. It could have discussed the clinical impact of introducing a novel test (minor, but positive improvement of health outcomes such as stoke and diabetes), but these were minor findings compared to the headroom analysis and aren't necessary for inclusion. *Thank you for your suggestion. We've incorporated more discussions about the clinical impact of introducing a novel test in 4.3.*
Perhaps remove the ':' symbol from the title of 4.1. *Corrected. Thanks!*
Good implications and future thinking, there are perhaps some caveats on where and how a test will be used in the care pathway that might impact this headroom - replacement test rather than add-on, earlier or later testing, intended population and inclusion criteria etc, but within a typical biomarker implementation pathway this framework provides a good overview of the general headroom available for novel diagnostics. *Thank you for your suggestion. We've incorporated more discussions about the alternative use of biomarker tests in GCA diagnosis in 4.3.*
Input parameter table is complete and well referenced.
Overall a well written paper, some minor typos, but factually complete. Nice, simple, uncomplicated decision model and clear transition model. Reasonable discussion based on the results identified. This article would add value for researchers, healthcare professionals and industry partners involved in biomarker development in giant cell arteritis. *Thank you for your kind comments.*

Reviewer: 2
Dr. Sarah Girdwood, FIND
Comments to the Author:
Summary
This study presents an early-stage cost-utility analysis to evaluate the potential value of hypothetical diagnostic biomarker tests for Giant Cell Arteritis (GCA). Using a combined decision-tree and Markov modelling approach, the analysis estimates ICERs and maximum cost-effective prices across varying sensitivity and specificity levels, compared to current diagnostic pathways. Results indicate that a rule-out test (confirmatory test) with high specificity to reduce false positives and thereby limit unnecessary glucocorticoid exposure is particularly valuable given the toxicity of continued empiric treatment.
• Section 2.1: In the Methods section, the authors refer to conducting a "targeted systematic review of economic literature" and a "targeted systematic search." This terminology is misleading, as no systematic review was conducted. The TABUL study plays a central role in shaping the model structure and parameter selection in this analysis. Given its influence, it would be more appropriate to introduce and contextualize the TABUL study earlier, ideally in the Introduction, rather than presenting it only in the Methods. Section 2.1 might be better titled "Model design and structure",

with a clear explanation of how the model was adapted or extended from the TABUL framework, and which parameters were drawn directly from it.
o Additionally, the rationale for limiting the search to studies with "economic evaluation" keywords is unclear. Many key parameters for the model are not economic in nature so this restriction may have led to the exclusion of relevant studies. Similarly, the decision to restrict the literature to UK-based studies is somewhat limiting. While UK data are appropriate for costing and population-level inputs, diagnostic performance data from non-UK settings could still provide valuable insights, particularly where UK data are limited or outdated.
o The following sentence doesn't feel necessary, nor do the authors need to justify it. "Due to resource limitations, we focused on identifying appropriate data sources rather than conducting a meta-analysis for each input parameter." Rather perhaps authors can just say they prioritised the use of published literature and expert opinion over formal meta-analyses.

*Thank you for your comments. While the TABUL study was helpful in informing certain aspects of our model, specifically the long-term extrapolation structure and diagnostic performance data for the standard test pathway, it does not form the central basis of our modelling approach. Our study is an early-stage economic evaluation focused on hypothetical biomarker tests for GCA diagnosis, and the model was independently developed for this purpose.*

*To support model development, we conducted a targeted literature review using a systematic search approach, albeit on a smaller scale than a full systematic review. The aim was to identify relevant sources for model structure and parameterisation. While the TABUL study was the only economic evaluation identified for GCA diagnostics, we did not restrict our search to UK-based studies and included international sources where appropriate, as well as for clinical and epidemiological inputs. However, as this review was not the focus of our analysis, we have now removed the section from the manuscript and simply highlighted the most relevant TABUL study we identified from the review.*

*We have also revised the manuscript to introduce the TABUL study in the Introduction and updated Section 2.1 (now titled 'Model overview') to clarify which elements were adapted from TABUL. Additionally, we have rephrased the sentence regarding resource limitations to more neutrally state that published literature and expert opinion were prioritised over formal meta-analyses.*

• Please clarify why a 19-month glucocorticoid duration was assumed. Indicate whether this reflects guidelines or typical prescribing practice, and note that glucocorticoids are not usually used long-term for GCA. *The 19-month glucocorticoid duration was based on the dosing schedule recommended in the 2020 British Society for Rheumatology (BSR) guideline for the management of GCA. This guideline outlines a tapering regimen*

*that typically concludes within 19 months, assuming no relapse or complications. We have now clarified this in the manuscript to reflect that the assumed duration is guideline-based rather than representative of long-term prescribing practice.*

o Also, consider commenting on how long AE risks persist after stopping treatment and whether these risks are assumed to decline over time in the model. *Unfortunately, we did not have access to individual patient-level data to examine the precise duration or trajectory of AE risks following cessation of glucocorticoid treatment. In our base case analysis, we relied on published odds ratios derived from statistical analyses of individual-level data from the Clinical Practice Research Datalink (CPRD). We assumed that new glucocorticoid-related complications would occur only within the first 5 years of treatment in the base case analysis. To explore uncertainty around this assumption, we conducted scenario analyses varying the duration of glucocorticoid-related condition impacts on costs and QALYs to 2 and 10 years. These analyses help capture the potential variability in AE persistence and its influence on model outcomes.*

• Clarify terminology for international readers—for example, briefly explain abbreviations for PSSRU and briefly describe what the PSS perspective includes. *Thank you for the suggestion. We have now added brief explanations of both the PSSRU and the PSS perspective in the Methods section, along with appropriate references. These clarifications aim to improve accessibility for international readers unfamiliar with UK-specific health economic terminology.*

• Consider using a starting age of 50, as GCA typically affects individuals aged 50 and older. While 71 reflects the TABUL cohort average, modeling from age 50 may better align with clinical onset and would likely increase headroom for cost-effectiveness. *Thank you for your suggestion regarding the starting age for the model. We agree that GCA typically affects individuals aged 50 and older, and we appreciate the rationale for considering an earlier starting age to potentially increase headroom for cost-effectiveness. In response, we conducted a sensitivity analysis using a starting age of 51. Interestingly, the results showed a lower headroom price compared to our base case. This appears to be due to the lower baseline mortality at age 50, which reduces the impact of excess mortality associated with steroid use. As a result, the QALY differences between FPS and TNs are smaller, leading to reduced incremental health benefits of the biomarker test. Given that our base case (starting age 71) reflects the average age of GCA diagnosis in the UK and is consistent with the TABUL cohort, we have retained it for the main analysis. However, we have included the age 51 scenario as a sensitivity analysis and discussed the implications in the manuscript.*

• Given the higher prevalence of GCA in women (e.g. 72% of study cohort is women in TABUL) and gender differences in life expectancy and risk profiles, this seems like a limitation to the study which could meaningfully affect results. The authors should either justify this approach more clearly or consider conducting a gender-stratified or weighted analysis, or at minimum, include a sensitivity analysis with female values for prevalence, life expectancy etc to assess its impact. *Thank you for the comment. We*

*have now adjusted the baseline mortality rates in the general population using a weighted average of female and male mortality rates, reflecting the higher proportion of women affected by GCA, as observed in the TABUL cohort. However, we were unable to conduct a fully gender-stratified analysis due to the lack of gender-specific risk estimates for glucocorticoid-related adverse events and GCA-related complications in the available data sources. We acknowledge this as a limitation and have noted it in the manuscript.*

• Performance

o Explicitly state that the model assumes a combined diagnostic accuracy based on biomarker tests plus existing blood tests (e.g. ESR, CRP), reflecting overall clinical decision-making. This should be stated explicitly, so manufacturers understand the performance target applies to the full algorithm, not the biomarker test alone. Otherwise, there's a risk of misinterpreting the required sensitivity and specificity, which may not be achievable by the biomarker in isolation. *Thank you for the suggestion. We have now revised the methods and results sections to emphasize the sensitivity and specificity values apply to the full diagnostic algorithm, not the biomarker test in isolation. This should help ensure that manufacturers interpret the performance targets appropriately.*

o In the TAB + US+ clinical judgement algorithm, wasn't TAB only conducted if US was positive? How was this sequential testing treated in the model? *Thank you for raising this point. In the TABUL study, the diagnostic performance for the TAB + US + clinical judgement pathway was derived from data where both TAB and US were conducted for trial participants. In our model, we did not explicitly account for sequential testing. Instead, as noted in the previous comment, we assessed the overall diagnostic performance of the full testing pathway, reflecting how clinical decisions are made in practice, based on a combination of symptoms, physical signs, laboratory tests, and confirmatory procedures. Therefore, for the standard pathway involving TAB + US + clinical judgement, we used the combined sensitivity and specificity values obtained when both tests were performed. While we acknowledge that, in current clinical practice, US is often used as the initial test and TAB is reserved for equivocal cases, many patients may undergo both investigations. In this analysis, we focused solely on the scenario where both tests were performed, as this was the only setting for which reliable diagnostic accuracy data were available.*

• Consider relaxing the assumption of full clinician adherence to test results and treatment guidelines. In practice, clinicians may continue or stop glucocorticoids based on clinical judgment, even with negative/positive test results (Doctor does not test – uses clinical judgement). If no data are available, consider reducing adherence from 100% to a more realistic estimate in sensitivity analysis and show how it affects the price of the new hypothetical test. *Thank you for your comment regarding clinician adherence to test results and treatment guidelines. We agree that in clinical practice,*

*decisions around initiating or discontinuing glucocorticoid treatment may not always strictly follow test outcomes, and clinicians often rely on their judgment.*
*To address this, we conducted sensitivity analyses assuming partial adherence scenarios, where either 50% or 75% TNs are treated as if they were TPs. While we do not have empirical data on these proportions, the assumptions were informed by discussions with our clinical co-authors who are familiar with current diagnostic and treatment practices. These analyses demonstrate how varying levels of adherence can influence the cost-effectiveness of the biomarker test, particularly by reducing the incremental QALYs gained when TNs are unnecessarily treated.*

• Also, would a less invasive, faster test increase testing access. E.g. not everyone gets a TAB now, but if it is a non- or less invasive biomarker test, would confirmatory testing increase thereby increasing access and reducing empiric treatment duration?

o On a related note regarding the timing of the new diagnostic test: the current algorithm is within 2 weeks, how would an instant biomarker test improve outcomes? How long does this new algorithm with blood tests take? If shorter, does the model incorporate these benefits (namely, reduced empiric treatment duration)?

*Thank you for this insightful comment. We agree that a less invasive and faster biomarker test has the potential to improve access to confirmatory testing, particularly for patients who currently do not undergo TAB due to limited availability or clinical uncertainty. A rapid biomarker test could help overcome these barriers, enabling earlier diagnostic confirmation and reducing reliance on empiric glucocorticoid treatment. While our model did not explicitly incorporate time-to-test results, we have now added a statement in the Discussion section to reflect the potential for such a test to reduce unnecessary glucocorticoid exposure and improve patient outcomes. This strengthens the case for further development and investment in biomarker-based diagnostics for GCA.*

• Is there any reason to believe that GCA glucocorticoid AEs are at a lower rate to non-GCA glucocorticoid AEs. *Thank you for your comment. In our analysis, we used GCA-specific estimates for both the baseline probability of glucocorticoid-related adverse events and the hazard ratios associated with glucocorticoid exposure from previously published CPRD analyses. This approach ensures that the AE risks incorporated into the model reflect the experience of the GCA population rather than general glucocorticoid users.*

• Consider using “maximum cost-effective price” or define “headroom” on first use, as it may not be familiar to all readers. *Thank you. We’ve defined the headroom in the methods section now.*

• Consider including a cost-neutral price—the price at which the new test yields the same cost/QALY outcomes as standard care. This may be more realistic for reimbursement (tempering manufacturing expectations) and more useful for policymakers and manufacturers. This may be closer to the 22 pound biomarker test.
*Thank you for the suggestion. We agree that highlighting the cost-neutral point -- where*

*the new test yields equivalent cost and QALY outcomes to standard care -- is important for informing realistic pricing expectations. In our model, this corresponds to a price of £0, as any additional cost would need to be offset by improved outcomes. However, we recognise the value of identifying the diagnostic performance level at which this equivalence occurs. We have now added this reference point to Figures 2a and 2b, allowing readers to see how small improvements in sensitivity or specificity (e.g. 1–2%) affect the maximum cost-effective price. This addition should help guide manufacturers and decision-makers in understanding the performance thresholds required for economic viability.*

• In the Discussion, it would be useful to have more of a discussion contextualising these results with regards to the TABUL study. How does this study add value to the TABUL study. How does it differ? Etc. *Thank you for the suggestion. While the TABUL assessed the cost-effectiveness of US compared to TAB for diagnosing GCA, our study evaluates the potential value of introducing a novel biomarker test. We used key input parameters from TABUL, such as the diagnostic performance of standard test pathways, but the research questions and scope differ. Our analysis focuses on early-stage economic modelling to inform test development, rather than comparing existing diagnostic strategies. Following previous suggestions, I have now introduced the TABUL study earlier and discussed clearly which part of our model followed the study.*

• Figure 2a and 2b have the same figure caption. Similarly, figure 3a and 3b. Add more detail around the range of sensitivity and specificity used so that they can be understood in isolation. *Thank you for the suggestion. The details have been added in the figure titles.*

• The purpose of Figures 3a and 3b is unclear. While they illustrate the QALY gains across varying test accuracy, these results are difficult to interpret without knowing what biomarker test price is assumed? If the goal is to show how small a QALY gain can still be cost-effective, this would need to be paired with the cost assumptions. Otherwise, Figures 2a/2b (headroom price) already capture the actionable trade-off between performance and price. Please consider clarifying the intent of these figures, as well as the biomarker price or removing them if they do not add interpretive value. *Thank you for the comment. Figures 3a and 3b were intended to illustrate the incremental QALY gains across varying levels of diagnostic accuracy, highlighting the potential health benefits of introducing a biomarker test. It does not need to rely on any cost assumptions. To avoid confusion, we have now added more explanations about these two figures and moved them to the supplementary materials.*

• Results: Would consider colour coding positive and negatives to make Table 2 easier to read. Are the numbers for vision loss across specificity correct (all zero)? *Thank you for your comment. We have now highlighted the negative values in Table 2 to improve readability. Regarding the vision loss outcomes, the values across varying specificity levels are indeed zero. This is because vision loss is a GCA-related complication rather than a steroid-related adverse event, and in our model, its risk is influenced by the*

*sensitivity of the diagnostic pathway (not specificity).*

• Results: There is no mention of the PSA? Was this conducted? *Yes, we conducted PSA to account for uncertainty in model parameters. As described in the Methods section, probability distributions were assigned to each uncertain parameter, and the model was run 5,000 times with values sampled from these distributions. The resulting mean and 95% CIs for maximum cost-effective prices and incremental QALYs are reported in the figures. To improve clarity, we have now added a statement in the Results section to explicitly reference the PSA and its role in generating the CIs.*

---

**VERSION 2 - REVIEW**

---

| | |
|---|---|
| **Reviewer** | **2** |
| **Name** | **Girdwood, Sarah** |
| **Affiliation** | **FIND, Impact** |
| **Date** | **01-Oct-2025** |
| **COI** | |

---

Thank you for responding to our my comments. It has certainly made the paper. I have only two minor clarifications/comments below:

• 1. Initial comment: Consider relaxing the assumption of full clinician adherence to test results and treatment guidelines. In practice, clinicians may continue or stop glucocorticoids based on clinical judgment, even with negative/positive test results (Doctor does not test – uses clinical judgement). If no data are available, consider reducing adherence from 100% to a more realistic estimate in sensitivity analysis and show how it affects the price of the new hypothetical test. Thank you for your comment regarding clinician adherence to test results and treatment guidelines. We agree that in clinical practice, decisions around initiating or discontinuing glucocorticoid treatment may not always strictly follow test outcomes, and clinicians often rely on their judgment.

Response: To address this, we conducted sensitivity analyses assuming partial adherence scenarios, where either 50% or 75% TNs are treated as if they were TPs. While we do not have empirical data on these proportions, the assumptions were informed by discussions with our clinical co-authors who are familiar with current diagnostic and treatment practices. These analyses demonstrate how varying levels of adherence can influence the cost-effectiveness of the biomarker test, particularly by reducing the incremental QALYs gained when TNs are unnecessarily treated.

Follow up comment: While TN-based non-adherence is arguably the most policy-relevant to model,real-world clinical behavior may also affect the management of TPs and FNs. It would

be helpful for the authors to briefly justify why their non-adherence sensitivity analysis focuses solely on TNs

2.• Initial comment: The purpose of Figures 3a and 3b is unclear. While they illustrate the QALY gains across varying test accuracy, these results are difficult to interpret without knowing what biomarker test price is assumed? If the goal is to show how small a QALY gain can still be cost-effective, this would need to be paired with the cost assumptions. Otherwise, Figures 2a/2b (headroom price) already capture the actionable trade-off between performance and price. Please consider clarifying the intent of these figures, as well as the biomarker price or removing them if they do not add interpretive value.

Response: Thank you for the comment. Figures 3a and 3b were intended to illustrate the incremental QALY gains across varying levels of diagnostic accuracy, highlighting the potential health benefits of introducing a biomarker test. It does not need to rely on any cost assumptions. To avoid confusion, we have now added more explanations about these two figures and moved them to the supplementary materials.

Follow-up comment: The caption appears to be wrong then as it states it is the incremental QALY at which the biomarker is cost-effective (i.e. there is an implicit assumption around costs)? "Figure B1a: Incremental QALYs at which the biomarker test is cost-effective at each diagnostic sensitivity (91-100%) and specificity (0-100%) pair for the biomarker test vs. standard test pathway of TAB and clinical judgment "

---

## VERSION 2 - AUTHOR RESPONSE

Reply to reviewer 2:
1) Thank you for your helpful follow-up comment regarding clinician adherence. We focused our sensitivity analyses on TNs, as this represents the most policy-relevant deviation from guideline-adherent practice, specifically the potential overtreatment of patients without GCA. We did not model non-adherence for TPs and FNs for the following reasons: TPs are typically treated following a positive test result, and our clinical collaborators noted that it is rare for clinicians to disregard such results unless a clear alternative diagnosis emerges. For FNs, patients are often re-evaluated based on worsening symptoms, and most are identified within a short timeframe (approximately four months), limiting the impact on long-term outcomes. We have now added a brief justification for this focus in the the last paragraph of 2.5.

2) Thank you for pointing this out. We agree that the original caption may have been misleading. We have now revised the figure captions as suggested.

---
